# Direct Determination of the Rate of Intersystem Crossing in a Near-IR Luminescent Cr(III) Triazolyl Complex

**DOI:** 10.1021/jacs.3c01543

**Published:** 2023-05-24

**Authors:** Robert W. Jones, Alexander J. Auty, Guanzhi Wu, Petter Persson, Martin V. Appleby, Dimitri Chekulaev, Craig R. Rice, Julia A. Weinstein, Paul I. P. Elliott, Paul A. Scattergood

**Affiliations:** † Department of Chemistry, University of Huddersfield, Queensgate, Huddersfield HD1 3DH, U.K.; ‡ Department of Chemistry, University of Sheffield, Brook Hill, Sheffield S3 7HF, U.K.; § Division of Theoretical Chemistry, Department of Chemistry, Lund University, Box 124, SE-22100 Lund, Sweden

## Abstract

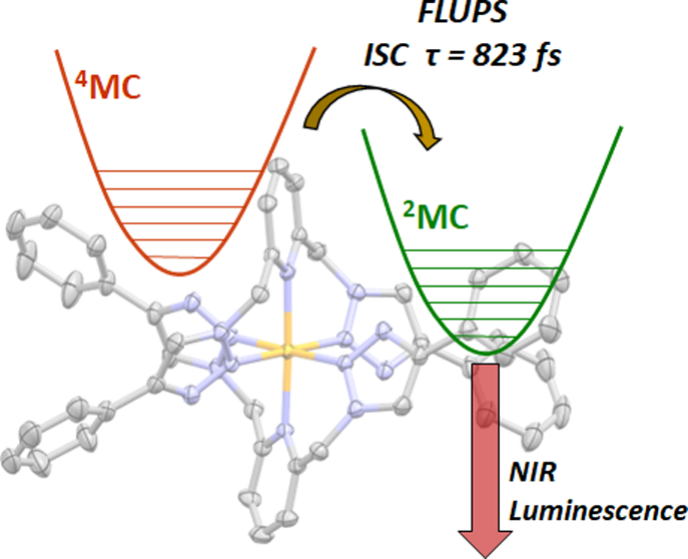

A detailed understanding of the dynamics of photoinduced processes occurring in the electronic excited state is essential in informing the rational design of photoactive transition-metal complexes. Here, the rate of intersystem crossing in a Cr(III)-centered spin-flip emitter is directly determined through the use of ultrafast broadband fluorescence upconversion spectroscopy (FLUPS). In this contribution, we combine 1,2,3-triazole-based ligands with a Cr(III) center and report the solution-stable complex [Cr(btmp)_2_]^3+^ (btmp = 2,6-*bis*(4-phenyl-1,2,3-triazol-1-yl-methyl)pyridine) (**1**^**3+**^), which displays near-infrared (NIR) luminescence at 760 nm (τ = 13.7 μs, ϕ = 0.1%) in fluid solution. The excited-state properties of **1**^**3+**^ are probed in detail through a combination of ultrafast transient absorption (TA) and femtosecond-to-picosecond FLUPS. Although TA spectroscopy allows us to observe the evolution of phosphorescent excited states within the doublet manifold, more significantly and for the first time for a complex of Cr(III), we utilize FLUPS to capture the short-lived fluorescence from initially populated quartet excited states immediately prior to the intersystem crossing process. The decay of fluorescence from the low-lying ^4^MC state therefore allows us to assign a value of (823 fs)^−1^ to the rate of intersystem crossing. Importantly, the sensitivity of FLUPS to only luminescent states allows us to disentangle the rate of intersystem crossing from other closely associated excited-state events, something which has not been possible in the spectroscopic studies previously reported for luminescent Cr(III) systems.

## Introduction

Cr(III)-centered coordination complexes have long been known for their wealth of photophysical and magnetic properties in addition to rich redox chemistry.^1−6^ However, there has been considerable renewed interest in photoactive complexes of this metal ion, driven in part through potential applications in photocatalysis,^7−13^ light-conversion systems,^14−17^ luminescence sensing,^18−20^ and as biological imaging agents.^21^ With such applications being presently dominated by the use of photoactive complexes of the rare and expensive 4d and 5d metal ions such as Ru(II), Os(II), and Ir(III), replacements based upon Earth-abundant, inexpensive, and more sustainable alternatives are highly attractive.^22−25^

The excited-state landscape in pseudo-octahedral complexes of Cr(III) is dominated by low-lying metal-centered (MC) excited states.^1,25−27^ Photoexcitation results in the population of ^4^MC states (e.g., ^4^T_2g_), the energy of which is dictated by the strength of the ligand field, and whose geometry is strongly Jahn–Teller distorted owing to the (t_2g_)^2^(e_g_*)^1^ electronic configuration.^2,25,27^ Prolonged population of these quartet excited states is often undesirable owing to their tendency to undergo ligand-substitution reactions. At sufficiently high ligand-field strength, two intraconfigurational and nondistorted doublet states (^2^T_1g_ and ^2^E_g_) are the lowest-lying excited levels and are readily populated by intersystem crossing (ISC) from the quartet manifold. When the ^4^T_2g_/^2^E_g_ energy gap is sufficiently large to prevent back-intersystem crossing (bISC), population of these states results in particularly long-lived photoluminescence in the deep-red and near-infrared (NIR) spectral regions.^6,23,24,28−30^ These nested ^2^MC states can also be strongly photooxidizing, displaying excited-state reduction potentials of up to +2 V vs NHE.^31^ Consequently, it can be seen that the rapid intersystem crossing process is central to achieving both stable and efficient photoactive Cr(III) complexes, the accurate experimental measurement of which is addressed in this work.

**Scheme 1:**
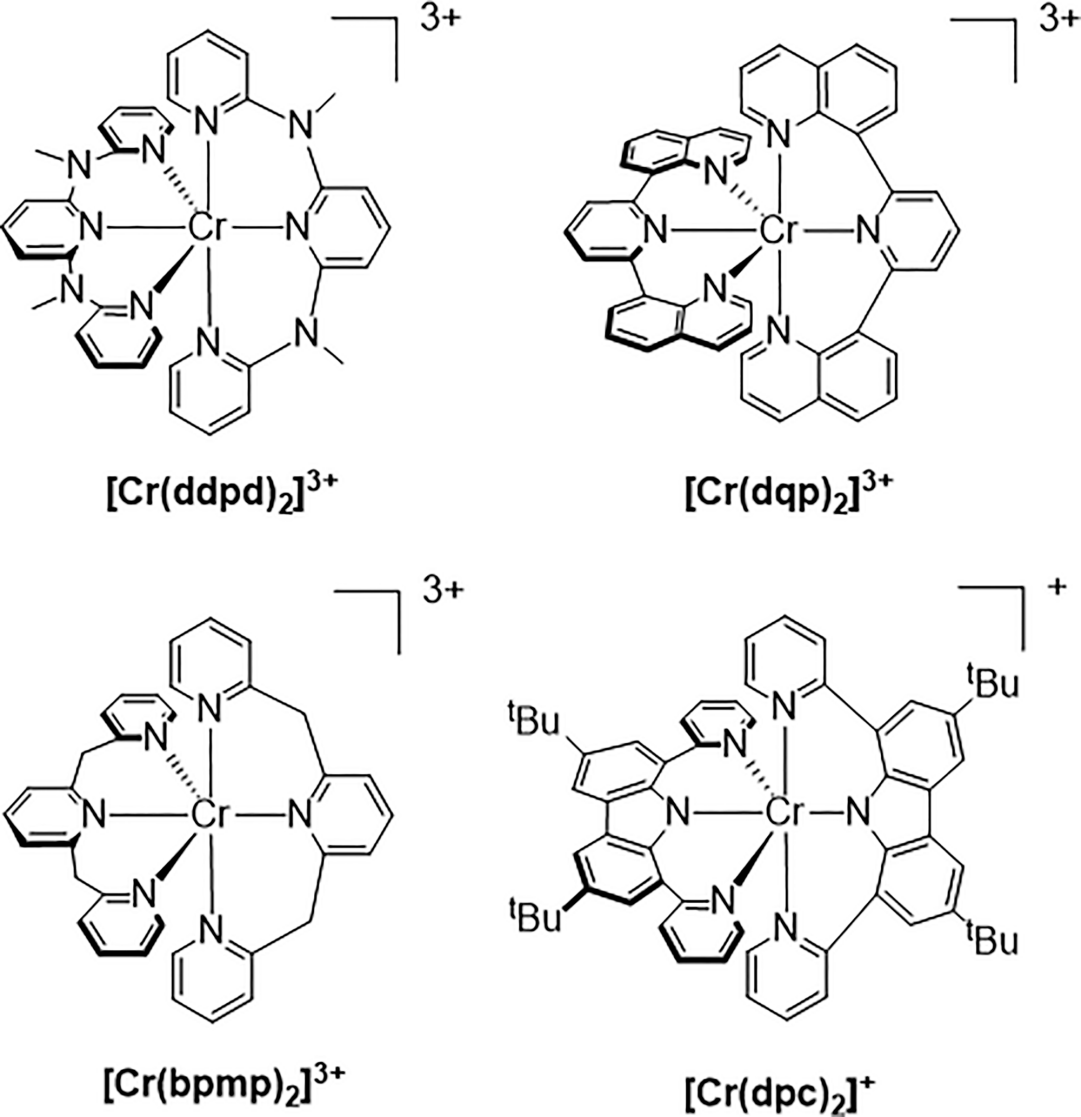
Molecular Structures of Selected Photoluminescent Cr(III) Complexes

While Cr(III) coordination complexes are undoubtedly attractive for use within light-driven applications, their widespread usage has been thus far precluded largely due to poor photoluminescence efficiencies. For example, while luminescence from the archetypal polypyridyl complexes [Cr(bpy)_3_]^3+^ and [Cr(tpy)_2_]^3+^ (bpy = 2,2′-dipyridyl; tpy = 2,2′:6′,2″-terpyridyl) can be detected and is fairly long-lived, it is exceptionally weak (ϕ < 0.01%).^6,32^ This is largely attributed to the aforementioned bISC owing to insufficient ligand-field strength, which in the case of [Cr(bpy)_3_]^3+^ is further facilitated by enhanced surface crossing as a result of trigonal distortions in the coordination sphere imparted by the five-membered chelates. A more recent and highly successful approach to molecular design involves the use of six-membered *tris*-chelates featuring strong-field donors which provide a near-perfect octahedral coordination environment around the metal center. The complex [Cr(ddpd)_2_]^3+^ (ddpd = *N*,*N*′-dimethyl-*N*,*N*′-dipyridine-2-ylpyridine-2,6-diamine)^33,34^ (Scheme 1) displays significantly enhanced photoluminescence (λ = 775 nm, ϕ = 13.6%) and a substantially increased excited-state lifetime (τ = 1.1 ms), while selective deuteration of the ligands further extends luminescence lifetime to 2.3 ms and achieves a record quantum yield for photoluminescence (ϕ = 30%).^35^ Likewise, the reported [Cr(dqp)_2_]^3+^ (dqp = 2,6-di(quinolin-8-yl)pyridine) (Scheme 1) displays strong deep-red luminescence (λ = 747 nm, ϕ = 5.2%) with an impressively long lifetime (τ = 1.2 ms) in aqueous solution.^36^ Interestingly, the nonplanar helical conformation adopted by the dqp motif upon coordination results in circularly polarized luminescence with a notably high dissymmetry factor (*g*_lum_ = 0.2).^36^ The dqp ligand also features in luminescent, although lesser explored, heteroleptic complexes of Cr(III) where it has been combined with both tpy and ddpd ligands in forming a series of complexes which demonstrate the ability to exert a degree of control and fine-tuning over both the lifetime and quantum yield of the observed luminescence.^37^ Use of a close analogue of ddpd featuring methylene bridges between the pyridyl donors, bpmp (bpmp = 2,6-*bis*(2-pyridylmethyl)pyridine) (Scheme 1) results in a complex displaying strong luminescence solely within the red portion of the visible spectrum, [Cr(bpmp)_2_]^3+^ (λ = 709 nm, τ = 1800 μs, ϕ = 19.6% in deaearted acidified D_2_O), with selective deuteration of the ligand raising the luminescence quantum yield further to ϕ = 25%, far surpassing the performance of classic red luminophores based upon Ru(II) and Eu(III).^38^ Two very recent reports have also made use of six-membered *tris*-chelate ligand architectures featuring a carbazolato donor fragment.^39,40^ Although [Cr(dpc)_2_]^+^ (dpc = 3,6-di-*tert*-butyl-1,8-di(pyridin-2-yl)-carbazolato) (Scheme 1) is only luminescent at cryogenic temperatures, the shift in position of phosphorescence into the NIR-II region (λ_em_ = 1067 nm)^39^ as a consequence of a much increased nephelauxetic effect represents an impressive step forward in the ability to tune the energy of emission in these systems.

As the usage of photoactive complexes of Cr(III) becomes more viable, further developments demand the need for robust and flexible ligand design and a move beyond the often employed pyridyl-based architectures. 1,2,3-Triazole donors are highly promising in this regard owing to the convenience of their preparation through copper-catalyzed click-chemistry and the ease in which substituents may be introduced at the 1- and 4-positions. These heterocyclic motifs present an ideal opportunity to append, for example, light-absorbing molecular antennae or biologically relevant residues to the periphery of a complex. Such functionalization may be particularly relevant to complexes of Cr(III) owing to their often very poor optical absorption within the visible region^41^ and the characteristic low-energy luminescence occurring in the biologically transparent region having implications toward uses within biological imaging. Furthermore, 1,2,3-triazole donors introduce flexibility into molecular design by offering variable modes of coordination through either the N(2) or N(3) positions depending upon the ligand structure.^42−45^ Although a singular report has been disclosed of a heteroleptic Cr(III)-centered ethylene polymerization catalyst featuring a fused benzotriazol-1-yl fragment,^46^ to the best of our knowledge, 1,2,3-triazole-based complexes of Cr(III) are unknown. This is perhaps a little surprising given the aforementioned flexibility offered by these donors and the wealth of reports which detail the rich photophysical and photochemical properties of complexes of these ligands.^47,48^

In the present study, we diversify the coordination chemistry of Cr(III) through the use of 1,2,3-triazole donors, arriving at a solution- and photostable complex which displays NIR luminescence with a lifetime on the microsecond timescale, a moderately high quantum yield and resulting high yield of ^1^O_2_ sensitization. We also probe the events occurring throughout the photoexcited landscape and their associated dynamics, paying particular attention to the all-important process of intersystem crossing which results in the desired ^2^MC states. While this very rapid spin-flip process has been detected in prior studies by transient absorption spectroscopy,^38^ particularly those concerning Cr(acac)_3_,^49,50^ the actual rate of ISC is difficult to extract owing to it being entangled with other excited-state processes (e.g., internal conversion, vibrational relaxation). Here, fluorescence upconversion spectroscopy allows us to selectively sample the very short-lived initially populated states in the quartet manifold and thus present a first example of direct determination of the rate of ISC in a Cr(III) complex.

## Results and Discussion

### Synthesis and Structural Characterization

**Figure 1:**
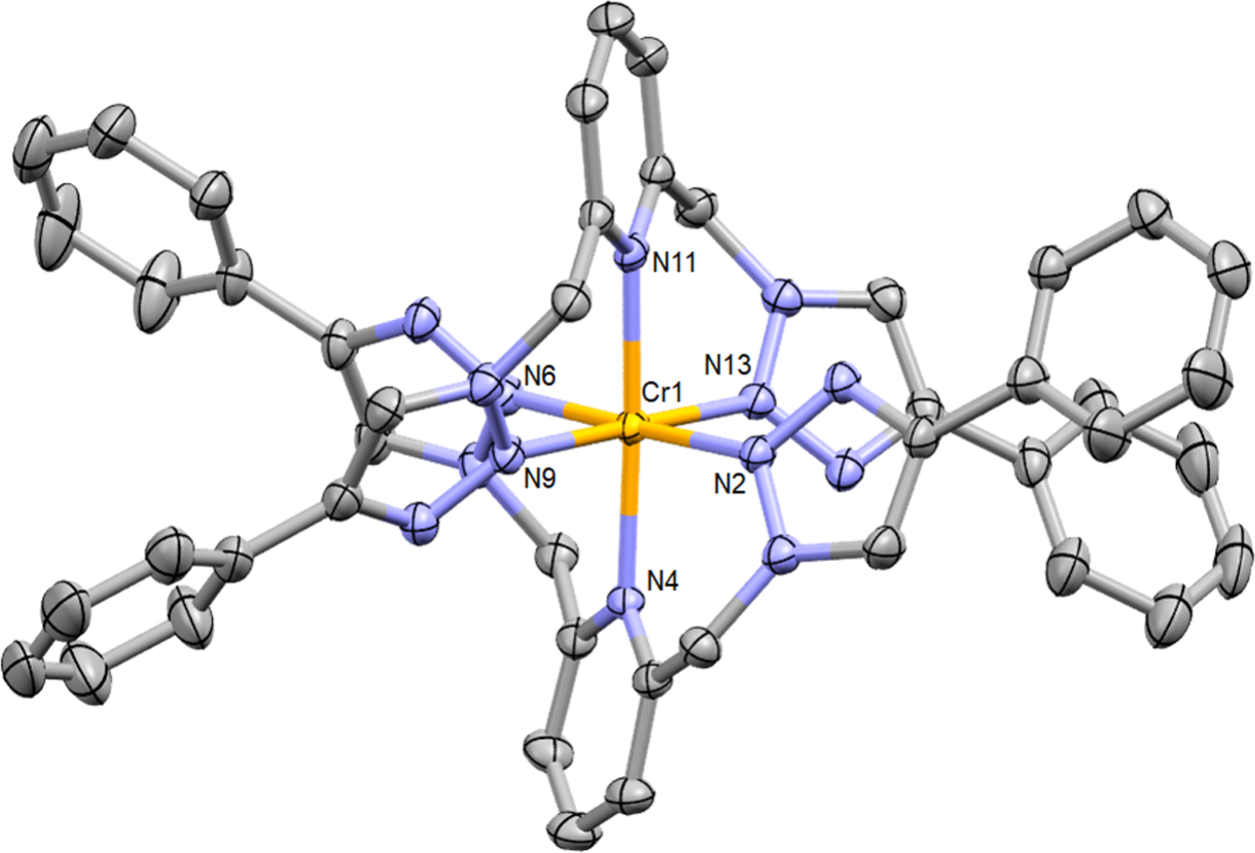
Molecular structure of [**1**^**3+**^][PF_6_]_3_. Thermal ellipsoids are shown at 50% probability, with hydrogen atoms, counterions, and co-crystallized solvent molecules removed for clarity. Selected bond lengths (Å) and angles (°): Cr(1)–N(2) = 2.017(2), Cr(1)–N(4) = 2.086(2), Cr(1)–N(6) = 2.021(2), Cr(1)–N(9) = 2.021(2), Cr(1)–N(11) = 2.090(2), Cr(1)–N(13) = 1.995(2); N(2)–Cr(1)–N(6) = 179.28(9), N(4)–Cr(1)–N(11) = 177.36(9), N(2)–Cr(1)–N(4) = 90.21(9), N(2)–Cr(1)–N(11) = 92.34(9), N(2)–Cr(1)–N(9) = 94.12(9) (CCDC = 2143431).

**Scheme 2:**
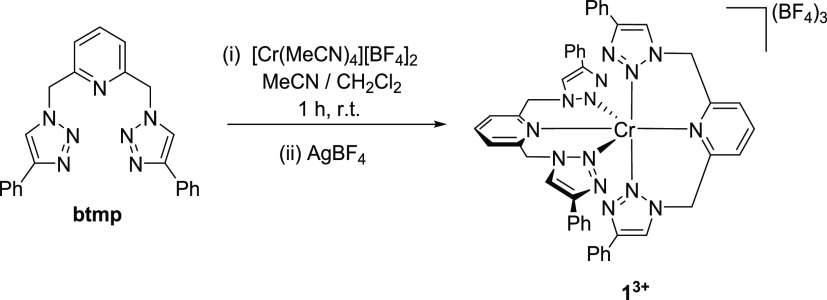
Molecular Structure of the Ligand btmp and the Synthetic Route to [**1**^**3+**^][BF_4_]_3_

Targeting a luminescent Cr(III) 1,2,3-triazole-based complex we selected 2,6-*bis*(4-phenyl-1,2,3-triazol-1-yl-methyl)pyridine (btmp) as a suitable ligand structure, with tridentate coordination through the central pyridyl and flanking triazole-N(2) donor atoms likely to provide a near-perfect octahedral coordination geometry. The synthesis of btmp has been previously reported,^51^ involving the one-pot CuAAC reaction of *bis*(bromomethyl)pyridine with phenylacetylene in the presence of NaN_3_, which in our hands proceeded smoothly with a yield of 93%. Mixing a solution of 2 equivalents of btmp with [Cr(MeCN)_4_][BF_4_]_2_ at room temperature (Scheme 2) resulted in an almost instantaneous deep-green colored solution, which upon treatment with AgBF_4_ yielded the desired complex [Cr(btmp)_2_][BF_4_]_3_ ([**1**^3+^][BF_4_]_3_), isolated as a bright-yellow solid with a good yield of 73%. The identity of **1**^**3+**^ was confirmed through mass spectrometry and elemental analysis. A magnetic susceptibility of 3.83 μ_B_ was determined through Evans’ method^52^ in excellent agreement with that expected for a d^3^ coordination complex with a quartet electronic ground state (3.87 μ_B_). While we were able to crystallize **1**^**3+**^ as its BF_4_^–^ salt, the crystals obtained were consistently unsuitable for X-ray diffraction. Counterion metathesis to the corresponding PF_6_^–^ salt however allowed the growth of crystals of X-ray diffraction quality as orange-colored plates by the vapor diffusion of diisopropylether into a concentrated acetonitrile solution of [**1**^**3+**^][PF_6_]_3_, the molecular structure of which is shown in Figure 1. [**1**^**3+**^][PF_6_]_3_ crystallizes in the **P**-1 space group, exhibiting N(2)–Cr(1)–N(6) and N(2)–Cr(1)–N(4) bonds angles of 179.28(9) and 90.21(9)°, respectively, highlighting the near-perfect octahedral coordination geometry. The bond lengths between the triazole donors and the chromium center (Cr(1)–N(2) = 2.017(2) Å, Cr(1)–N(13) = 1.995(2) Å) are marginally shorter than those to the pyridyl moieties (Cr(1)–N(4) = 2.086(2) Å, Cr(1)–N(11) = 2.090(2) Å), which themselves are only slightly longer than those to the central pyridyl donor in related complexes such as [Cr(ddpd)_2_][PF_6_]_3_ (2.05 Å)^34^ and [Cr(bpmp)_2_][OTf]_3_ (2.06 Å).^38^ The X-ray crystal structure also confirms the chelation-driven coordination of the triazole moieties through the less basic N(2) donors. Coordination through the N(3) positions is not observed as this precludes the formation of chelate rings with the central pyridyl donor.^42,44,51,53^ The btmp framework is nonplanar, adopting a buckled conformation which results in a helical wrapping around the metal center akin to that observed in Cr(III) complexes of ddpd^34^ and dqp.^36^

### Photophysical and Electrochemical Properties

**Figure 2:**
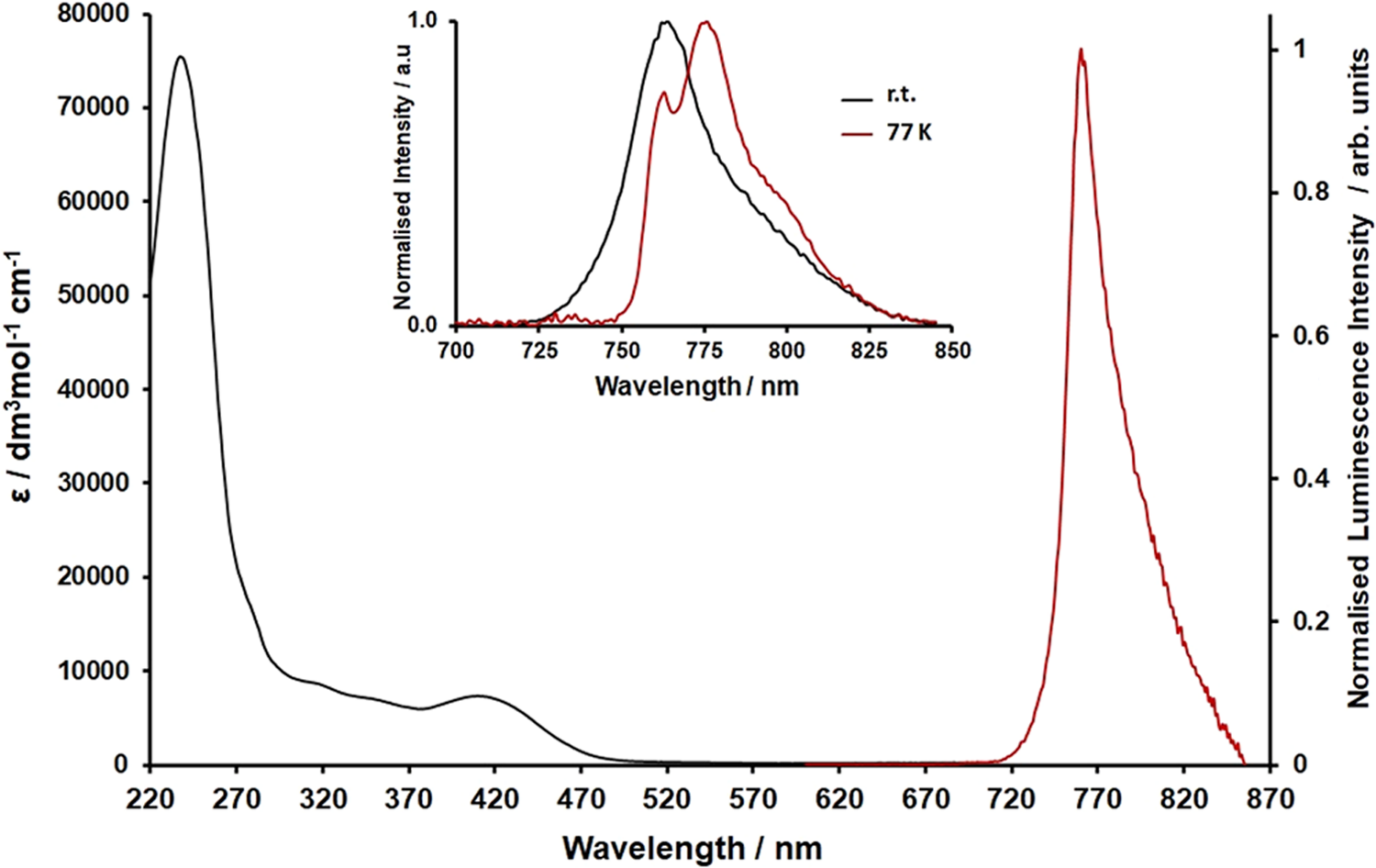
UV–visible electronic absorption (black) and photoluminescence (λ_ex_ = 450 nm, red) spectra recorded for an aerated acetonitrile solution of [**1**^**3+**^][BF_4_]_3_. Inset: photoluminescence spectra recorded for a room-temperature solution of [**1**^**3+**^][BF_4_]_3_ in 4:1 EtOH/MeOH (black) and at 77 K in a 4:1 EtOH/MeOH solvent glass (red) (λ_ex_ = 445 nm).

The UV–visible electronic absorption spectrum of **1**^**3+**^ in acetonitrile is shown in Figure 2. Intense absorption bands between 220 and 300 nm are assigned to ligand-localized π → π* transitions, supported by the occurrence of these features within the absorption spectrum of the free ligand (Figure S4). The broad low-energy absorption feature centered at 410 nm is assigned to a mixture of ligand-to-metal charge transfer excitations with contributions from spin-allowed ligand-field transitions of ^4^A_2_→^4^T_2_ character, owing to the latter being typically weak (ε < 500 dm^3^ mol^–1^ cm^–1^). This assignment is supported through TD-DFT calculations, where the 10 lowest vertical excitations of quartet multiplicity consist of a set of narrowly spaced transitions of both LMCT and MC character (vide infra and Supporting Information). Those excitations of predominantly MC character, having the expected very low oscillator strengths, are positioned at the low-energy edge of the calculated absorption band and are thus likely to be responsible for the very weak tail observed in the experimental spectrum beyond 500 nm. Further, the moderately weak absorption envelope between 300 and 380 nm is ascribed to a combination of further ligand-field and LMCT transitions, in addition to weak spin-forbidden ligand-localized π^1^ → π^3^* excitations. Transitions of metal-to-ligand charge transfer (MLCT) character are ruled out owing to the Cr(III/IV) couple being thermodynamically inaccessible and with the btmp ligand being difficult to reduce. This is evidenced through cyclic voltammetry, where no oxidation processes are observed within the available electrochemical solvent window, with only a cathodic metal-centered reduction wave being recorded at *E*_p_^c^ = −2.48 V vs Fc^+^/Fc (Figure S5). While previous reports have highlighted that reduction processes within Cr(III) complexes can be either ligand-^54,55^ or metal-centered,^34^ DFT calculations for the one-electron reduced complex **1**^**2+**^ (Figure S17) find the metal-centered quintet state (^**5**^**1**^**2+**^) to be significantly lower in energy than the corresponding triplet state (^**3**^**1**^**2+**^) and suggest that the reduction process observed here is indeed predominantly localized on the Cr(III) center.

When excited at 450 nm, **1**^**3+**^ is luminescent in aerated acetonitrile solution (Figure 2 and Table 1), displaying a narrow, featureless band with λ_max_ = 760 nm, which is typical for Cr(III)-centered emitters^6,31,41,56,57^ and ascribed to emission from a low-lying spin-flip ligand-field excited state of doublet multiplicity. The excitation profile closely follows the electronic absorption spectrum between 280 and 500 nm (Figure S6), suggesting that the luminescent doublet states are efficiently populated from both ^4^LMCT and ^4^MC states. However, deviation of the excitation profile from absorbances below 280 nm suggests that this is not necessarily the case for the higher-energy, predominantly ligand-centered excited states in this complex.

**Table 1:** Summarized Photoluminescence Data for Room-Temperature Solutions of [**1**^**3+**^][BF_4_]_3_

solvent	λ_max_, nm^a^	Φ_em_, %^b^^,^^d^	Φ_em_, %^c^^,^^d^	τ_em_, μs^b^	τ_em_, μs^c^
MeCN	760	0.11	0.27	13.7	37.9
*d*_3_-MeCN	760	0.11	0.46	13.8	51.3
H_2_O	760	0.23	0.42	26.3	57.1
D_2_O	760	0.23	0.59	24.5	55.7
MeCN/0.1 M HClO_4_	760	0.13	0.54	13.5	41.5

a λ_ex_ = 450 nm.

b Aerated solution.

c Deoxygenated solution.

d Measured relative to [Ru(bpy)_3_]^2+^, ϕ = 1.8% in aerated MeCN, ϕ = 4.0% in aerated H_2_O.^58^

Photoluminescence of **1**^**3+**^ in aerated acetonitrile is weak (ϕ_em_ = 0.1%) although increases ∼2-fold under deaerated conditions with an accompanying elongation of emission lifetime from 13.7 to 37.9 μs (Table 1). The sensitivity of luminescence to molecular oxygen is well documented for complexes of Cr(III), arising due to energy transfer from the luminescent doublet excited states to ^3^O_2_.^13,59^ We thus proceeded to determine the quantum yield for singlet oxygen sensitization, arriving at a modest value of ϕ[^1^O_2_] = 47% which highlights the potential of complexes such as **1**^**3+**^ in photodynamic therapy^60^ and photocatalysis applications.^9,13^**1**^**3+**^ remains luminescent in aqueous solution, displaying an enhancement in both ϕ_em_ and τ_em_ relative to acetonitrile solution, while acidification of acetonitrile solutions results in a further small increase in photoluminescence quantum yield, reaching 0.5% under deaerated conditions (Table 1). Removal of high-frequency oscillators through deuteration of the solvent reduces the rate of nonradiative decay,^35^ raising τ_em_ in *d*_3_-MeCN by 1.3-fold and allowing ϕ_em_ = 0.6 % to be realized in deaerated D_2_O. Although **1**^**3+**^ far surpasses classical Cr(III) emitters such as [Cr(bpy)_3_]^3+^ (ϕ = 8.9 × 10^–2^ %),^6^ the photoluminescence attributes remain inferior to the new generation of “molecular-ruby” luminophores.^34^

Interestingly, **1**^**3+**^ displays dual emission in a solvent glass at 77 K (Figure 2, inset). Upon cooling, the band at 760 nm sharpens, displays a significantly increased lifetime (τ_em_^77 K^ = 782 μs), and is accompanied by the appearance of a second, more intense band at 776 nm featuring a clear shoulder at 800 nm. Such dual luminescence is typical for emissive Cr(III) complexes,^1,6,18,24,37,56,61^ albeit usually readily observed at room temperature, and describes a Boltzmann distribution between two closely spaced equilibrated doublet states. For **1**^**3+**^, the appearance of the lower-lying state at low temperature may indicate that the two emitting states differ in their respective radiative rates, or that the higher-lying of the two doublet states is less prone to thermally activated nonradiative decay channels and is thus the more emissive of the pair. This is likely to be a consequence of enhanced surface crossing between the lowest doublet excited state and the ground state, facilitated by a degree of structural distortion of the former relative to the latter. This is somewhat evidenced by the appearance of vibronic progressions associated with the low-energy band and is further supported by a similar observation having recently been made for the structurally similar complex [Cr(bpmp)_2_]^3+^ upon cooling to 10 K in a KBr matrix.^38^

While we clearly observe luminescence from two different doublet excited states in **1**^**3+**^, we have thus far refrained from assigning the parentage of these states. Over the last few decades, the literature concerned with luminescent polypyridyl complexes of Cr(III) consistently assigns the higher- and lower-lying of the two doublet states to be of ^2^T_1_ and ^2^E character, respectively.^31,32,41,56,57,61−63^ However, recently reported detailed computational studies have indicated that a micro-state of ^2^T_1_ parentage can drop below ^2^E levels and is likely to be origin of the lowest-energy emission band observed for “molecular-ruby class” complexes such as [Cr(dppd)_2_]^3+^ and [Cr(bpmp)_2_]^3+^.^19,38^ Given the structural similarity of **1**^**3+**^ to these molecular systems, we tentatively assign the higher- and lower-lying of the luminescent doublet states to be of ^2^E and ^2^T_1_ character, respectively. However, given the closeness in energy of these doublet states and the considerable complexities associated with a correct theoretical prediction of the exact ordering of excited states, we stress that this assignment must be treated with caution.

### Solution- and Photostability Studies

To assess the solution stability of **1**^**3+**^, samples of the complex in aerated acetonitrile and water (protected from light) were monitored by UV–visible absorption spectroscopy. The profile of spectra recorded for acetonitrile solutions over 12 h reveal negligible changes in absorption, remaining unchanged after a further 4 days (Figure S7). Aqueous solutions of **1**^**3+**^ on the other hand appear stable over 8–12 h but undergo small spectral changes over 72 h, suggesting that the complex may be susceptible to slow aquation upon prolonged dissolution in water (Figure S8). As Cr(III) polypyridyl complexes are known to undergo photoaquation reactions^5,64,65^ and with 1,2,3-triazole-containing ligands having been previously shown to induce interesting photochemical reactivity,^48^ we proceeded to examine the photostability of **1**^**3+**^ in both acetonitrile and water. Under irradiation with a 23 W compact fluorescent lamp which contains both UV and visible spectral components (see Figure S9 for output profile), acetonitrile solutions of **1**^**3+**^ undergo negligible changes in both their electronic absorption and photoluminescence spectra over a 2 h period (Figures S10 and S11), whereas spectra recorded for aqueous solutions reveal a loss of ∼50 % over a 70 min period (Figure S12). Under identical conditions, [Cr(bpy)_3_]^3+^ is completely consumed. As the rate of photoaquation is retarded in acidic media,^64^ photolysis experiments were repeated for aqueous 0.1 M HCl solutions. While [Cr(bpy)_3_]^3+^ was not completely photoaquated after 2 h, a steady decrease in absorption bands associated with **1^3+^** indicates ∼75 % consumption (Figure S13). As the previously proposed mechanism for photoaquation involves the deprotonation of coordinated water molecules,^5,41,64^ it is suggested that this step is facilitated by the presence of the basic, uncoordinated triazole-N(3) donors. This behavior, together with the weaker coordination of N(2)-bound triazolyl moieties over their pyridyl counterparts, is likely to be responsible for the observed photoreactivity of **1**^**3+**^ in aqueous solution. However, as **1**^**3+**^ exhibits excellent solution- and photostability in acetonitrile, we were able to proceed to examine the photophysical properties in more detail.

### Computational Studies

To complement our structural studies of **1**^**3+**^ and to assist in gaining a deeper insight from our photophysical studies (vide supra) we carried out quantum chemical calculations. Fully optimized geometries were first obtained by unrestricted density functional theory calculations (uDFT) for the lowest-energy quartet and doublet states of **1**^**3+**^ in implicit solvent (MeCN). The calculated ground state quartet geometry is in overall good agreement with that obtained through X-ray crystallography, indicating the buckled conformation of the ligand around the metal center and the essentially octahedral coordination environment. The calculated relative total energies of the two states confirm the expected significant preference for the quartet ground state by 1.97 eV. Calculated spin density plots as well as associated selected molecular orbital plots and calculated Mulliken spin densities on the Cr center (Figure S16 and Table S1) are consistent with electronic structural assignments of the quartet and doublet states as mainly having metal-centered open shells with some metal-ligand mixing.

In addition, single-point energies were calculated for the doublet excited state at the relaxed quartet ground state geometry and vice versa (Table S1) indicating small intrastate energy relaxations of 0.05 eV for each of the states and thus being consistent with the expected small potential energy surface distortions associated with an energy landscape characterized by nested metal-centered states.

Finally, some excited-state properties relating to the spectroscopic observations are considered through a combination of uDFT properties of the doublet state and vertical quartet–quartet excitations obtained from time-dependent density functional theory (TD-DFT) calculations, although it should be noted that the computational accuracy and reliability should be considered with caution for the open-shell ground and excited states. According to the TD-DFT calculations (see the Supporting Information) the lowest calculated vertical quartet–quartet excitations from the relaxed ground state geometry consist of a set of narrowly spaced excitations, the lowest of which is calculated at 2.18 eV (569.8 nm) and the first to have an oscillator strength exceeding 0.01 found at 2.40 eV (516.1 nm), in reasonable agreement with the experimental absorption threshold around 500 nm and the broad, weak appearance of the lowest-energy absorption feature. Upon further inspection, the excitations were found to be a mixture of LMCT and MC excitation configurations, with those of particularly low oscillator strength (*f* < 0.0005) in the region 2.17–2.30 eV being consistent with transitions where MC character dominates. Although the lowest-energy transitions appear to have a small degree of LMCT character, this configuration dominates the more intense excitations in the region of 2.40–2.47 eV, with the highest energy calculated vertical excitations revealing transitions composed of both LMCT and ligand-centered character.

A simple estimate for the lowest excited-state deactivation can furthermore be obtained from the vertical difference in total energy between the relaxed doublet excited state and the quartet ground state at the same geometry, which is calculated to be 1.92 eV at the uDFT level of theory. This corresponds to a wavelength of 646 nm, somewhat overestimating the experimental luminescence energy threshold at ca. 1.75 eV (710 nm). However, agreement to within 0.2 eV is not unreasonable given the uncertainties in reliably calculating excited-state energies for open-shell transition-metal systems without more rigorous methodological bench-marking and is not dissimilar to that recently reported for a combined experimental and theoretical study of a luminescent Cr(III) complex.^39^

### Ultrafast Transient Absorption Spectroscopy

**Figure 3:**
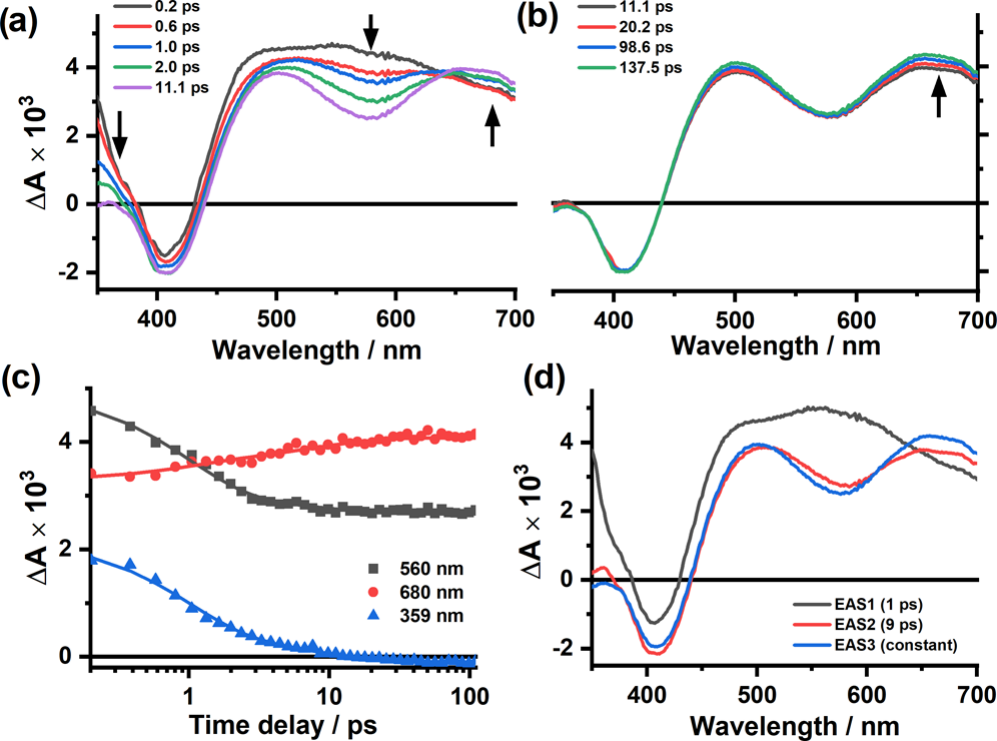
Transient absorption spectra of **1**^**3+**^ in aerated acetonitrile (λ_ex_ = 400 nm) showing details of transients recorded 250 fs to 11 ps (a) and 11–100 ps (b) following excitation; selected single-point kinetic traces obtained from global analysis (c); and evolution-associated spectra (EAS) extracted from global analysis (d) (black arrows indicate the direction of spectral change). The instrument response function was 100 fs (fwhm).

In order to further explore the excited-state behavior and dynamics of **1**^**3+**^, we carried out transient absorption experiments for an aerated acetonitrile solution (Figure 3). Following excitation at 400 nm, a transient signal rapidly evolves across the entire spectral window (420–700 nm), reaching a maximum within 250 fs and displaying a broad excited-state absorption (ESA) with maxima at 485 and 555 nm (Figure 3a). Accompanying the rise in ESA is a bleach feature at 407 nm corresponding to the depopulation of the ground state. Over the proceeding 10 ps, a rapid decay of ESA bands is observed, concomitant with an increase in electronic absorption between 630 and 700 nm. Global lifetime analysis (GLA) reveals that this process occurs with a time constant of 1.1 ps (τ_1_), with the appearance of an isosbestic point at 630 nm indicative of population transfer to a new excited state. Over the same time period, a slight deepening of the ground state bleach is observed, suggesting that this negative feature is partially overlapped by the ESA associated with the initially formed excited state. Further GLA allows deconvolution of the initial dynamics (0.25–11 ps) into two contributing evolution-associated spectra (EAS), the first corresponding to the species present at 250 fs and the second resulting from the decay with τ_1_ = 1.1 ps. These EAS are notably different from one another, with the former displaying a broad feature centered at 550 nm and the latter showing a comparatively narrowed band at 505 nm together with a low-energy absorbance peaking at 660 nm and extending further beyond 700 nm (Figure 3d).

Further subtle changes are then observed in the transient spectra over the following 100 ps, characterized by a marginal increase in the low-energy ESA between 600 and 700 nm (Figure 3b). The transient spectra across the remaining spectral range are unchanged, with the constant magnitude of the bleach feature indicating that subsequent dynamics (over the range 11–100 ps) do not correspond to re-population of the ground state. The evolution of these small changes is fitted with a time constant of τ_2_ = 9 ps, giving the third and final EAS which does not decay (τ_3_ = constant) and is considered quasi-stationary on the timescale of the experiment (8 ns). The second and third EAS are near-identical, inferring that τ_2_ is associated with relaxation within the same excited state or represents an equilibration between states of very similar energy and electronic structure.

From these experimental observations, we are able to assign the fast component (τ_1_ = 1.1 ps) to a combination of electron redistribution within, and intersystem crossing (ISC) from, the initially populated ^4^LMCT/^4^MC states to ^2^MC states. This assignment is supported by the clear and distinct nature of the first and second EAS and the anticipated significant differences in electronic structure between these states. Importantly, this time constant can only be treated as an approximation of the rate of ISC itself, as accompanying excited-state processes such as internal conversion (IC) and vibrational relaxation (VR) most likely make some contribution to the spectral changes observed over this time period. The second, slower process (τ_2_ = 9 ps) is ascribed to vibrational relaxation and thermal equilibration between the close-lying ^2^E and ^2^T_1_ metal-centered excited states, consistent with the very similar profiles of the second and third EASs. The quasi-constant final state is straightforwardly assigned to the thermally equilibrated and luminescent ^2^E and ^2^T_1_ levels, which have a lifetime on the microsecond timescale (vide supra) and an ESA somewhat reminiscent of those observed for [Cr(ddpd)_2_]^3+^,^13^ [Cr(phen)_3_]^3+^ (phen = 1,10-phenanthroline)^32^ and [Cr(dmcbpy)_3_]^3+^ (dmcbpy = 2,2′-bipyridine-4,4′-dicarboxylate).^31^

### Ultrafast Fluorescence Upconversion Spectroscopy

With the information gathered through transient absorption spectroscopy now in hand, we proceeded to probe the excited-state landscape and ultrafast dynamics of **1**^**3+**^ further, paying particular attention to the intersystem crossing process and the population of short-lived quartet excited states prior to ISC. We thus turned to ultrafast broadband fluorescence upconversion spectroscopy (FLUPS). This technique selectively reports on fluorescent excited states on the femtosecond timescale, therefore being ideally suited to monitor excited-state processes involving a change in spin state, allowing the dynamics of ISC to be disentangled from those of other nonradiative excited-state events (e.g., IC, VC), something which is difficult to achieve by other spectroscopic means.^66,67^ Indeed, fluorescence upconversion spectroscopy experiments have previously been used to good effect in determining the rate of ISC in the ubiquitous [Ru(bpy)_3_]^2+^, arriving at a value of τ = 15 ± 10 fs following the observation of short-lived fluorescence from the Frank–Condon ^1^MLCT state prior to ISC.^68^

**Figure 4:**
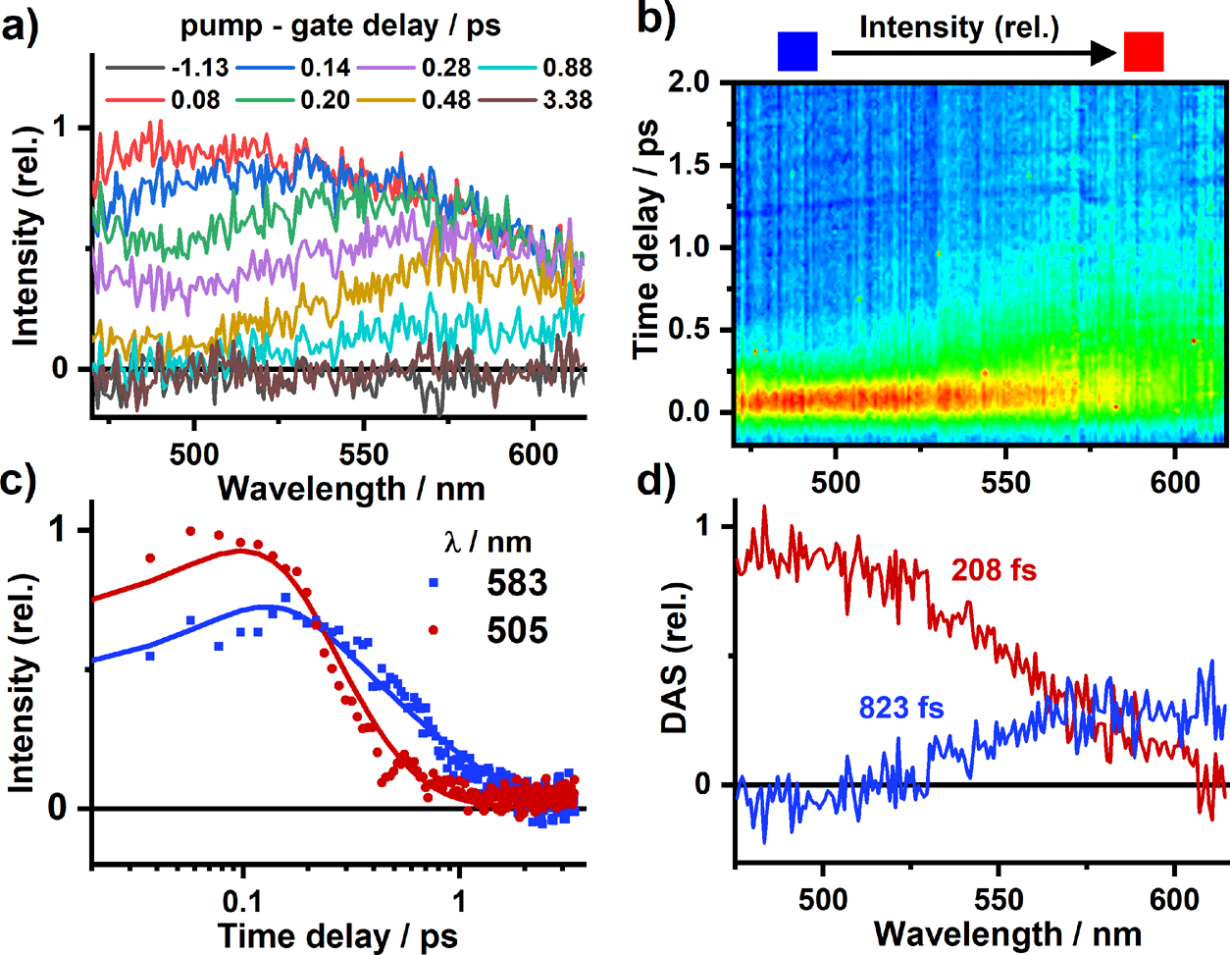
(a) fs Fluorescence upconversion spectra (FLUPS) recorded for an aerated MeCN solution of **1**^**3+**^ following excitation with 400 nm, 40 fs pulses; (b) FLUPS signal intensity map recorded at 2 ps after the excitation; (c) single-wavelength decay kinetics (symbols), with solid lines representing fit to the data using parameters obtained by global lifetime analysis (208 and 823 fs); (d) decay-associated spectra (DAS) obtained from global analysis (the instrument response function was 200 fs (fwhm)).

FLUPS spectra obtained for aerated acetonitrile solutions of **1**^**3+**^ following 400 nm, 40 fs excitation are shown in Figure 4. After deconvolution of instrument response (200 fs, fwhm across the spectral window), a broad, weak fluorescence band is detected across the 475–625 nm spectral window, being fully formed at the earliest detectable time delay (80 fs) following excitation (Figure 4a). Over the proceeding 1 ps the emission decays, with the intensity of the signal in the shorter-wavelength region decreasing faster than that at lower energies. These changes are more clearly represented in the decay-associated spectra (DAS) (Figure 4d) which reveal two fluorescence bands centered around 490 and 590 nm. Global lifetime analysis allows time constants of 208 and 823 fs to be extracted for the decay of these higher- and lower-energy emission bands, respectively.

In order to support these spectroscopic observations we turned to steady-state fluorescence spectroscopy, detecting two distinct but extremely weak bands in the region of 485 and 615 nm for an independent sample of **1**^**3+**^ in aerated acetonitrile solution (Figure S14). We were unable to determine luminescence lifetimes for either feature by time-correlated single photon counting, confirming fluorescence on the sub-nanosecond timescale. The excellent agreement between the steady-state spectra (Figure S14) and the two DAS obtained through FLUPS measurements (Figure 4d) confirm the population of two distinct short-lived luminescent excited states at the earliest detection time after the photoexcitation.

When attempting to identify these two different luminescent excited states, we note that the 400 nm excitation source is energetically competent in populating both ^4^LMCT and ^4^MC states which we earlier identified through TD-DFT calculations (vide supra and Supporting Information). These theoretical studies are of further value by indicating that, while there appears to be a degree of metal-ligand mixing, relaxed states of predominantly ^4^LMCT character lie ∼19 600 cm^–1^ above the quartet ground state, whereas ^4^MC levels are lower in energy in the region of 17 850 cm^–1^, aligning reasonably closely with the maxima of the emission bands observed by both FLUPS and steady-state fluorescence spectroscopy. Treated in conjunction with prior reports detailing the typical excited-state ordering in Cr(III) complexes^69^ and the extremely short emission lifetimes, these data allow us to make an initial assignment of the two luminescent states observed by FLUPS to be those of primarily ^4^LMCT and ^4^MC character, respectively.

Importantly, the observation of fluorescence from the ^4^MC state prior to deactivation through spin-flip and entry into the doublet manifold allows a direct measure to be made of the intersystem crossing process, deconvolved of any parallel nonradiative excited-state events, allowing us to arrive at an upper value of 823 fs. This time constant is very close to the 1.1 ps component extracted from transient absorption spectroscopy (vide supra) which was assigned to convolved ISC and electronic redistribution processes associated with decay of the ESA arising from the population of states within the quartet manifold. Alongside depopulation of the ^4^MC state, we notice that the higher-lying ^4^LMCT state is shorter-lived. This could, in part, be due to rapid internal conversion to the lower-lying ^4^MC state, but one cannot rule out faster ISC to higher-lying doublet states (e.g., ^2^T_2_) within 208 fs. It is plausible that the two spectroscopically observed quartet states may undergo differing rates of ISC due to the varying density of states which exist within the excited doublet manifold.

Compared to the previously reported Cr(III) systems, the timescale of ISC directly determined for **1**^**3+**^ is considerably longer than that estimated for [Cr(acac)_3_] (τ_isc_ = 50 fs) by femtosecond transient absorption spectroscopy.^49^ This is perhaps unsurprising given the obvious dissimilarities in ligand structure and consequently also in the electronic structure, density of states as well as vibrations potentially involved in the ISC process. However, the ISC process is only ∼1.5 times slower than that estimated for [Cr(bpmp)_2_]^3+^ (τ_ISC_ = 540 fs),^38^ consistent with the structural similarities between the btmp and bpmp ligand systems, the identity of the donor groups and consequent overall electronic structures of the resultant Cr(III) complexes.^38^ With these studies demonstrating that the rate of ISC may be directly determined through the use of FLUPS spectroscopy, it is possible that such measurements carried out for a wider range of Cr(III) systems may aid and further advance our understanding of structural parameters which govern this excited-state process.

## Conclusions

**Figure 5:**
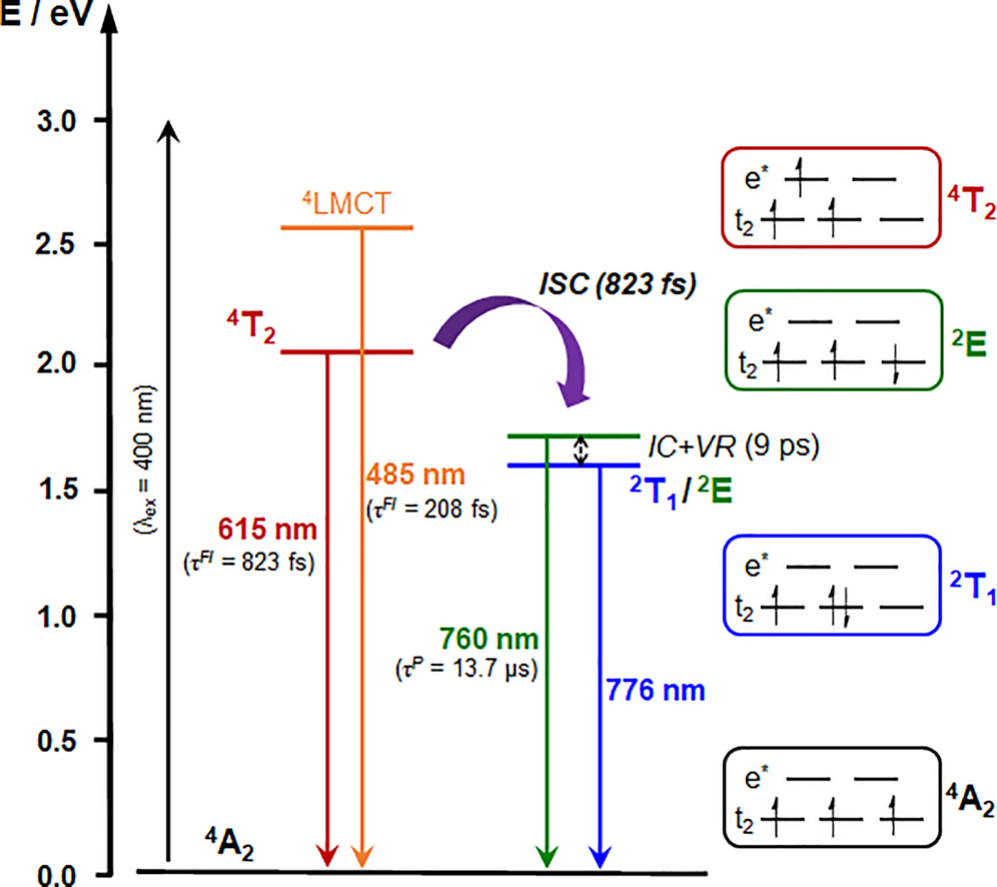
Schematic energy level diagram depicting the excited-state behavior of **1**^**3+**^ as determined through steady-state and time-resolved spectroscopies and theoretical calculations (ISC = intersystem crossing, IC = internal conversion, VR = vibrational relaxation).

We have now diversified Cr(III) coordination chemistry to include 1,2,3-triazole donors, leading to a solution- and photostable luminophore. Photoexcitation of aerated acetonitrile solutions of [Cr(btmp)_2_]^3+^ (**1**^**3+**^) results in NIR emission (λ_em_ = 760 nm) from a spin-flip doublet metal-centered excited state which exhibits a lifetime on the microsecond timescale. Photoluminescence lifetimes can be extended ∼3-fold upon excluding molecular oxygen, with a yield of singlet oxygen sensitization of 47% indicating the potential of this system for photocatalytic and photodynamic therapy applications. Ultrafast transient absorption spectroscopy elucidates the excited-state behavior and dynamics of **1**^**3+**^, revealing the luminescent doublet excited states to be formed within 1 ps following photoexcitation. Femtosecond fluorescence upconversion spectroscopy (FLUPS) allowed us to directly and accurately determine the rate of intersystem crossing between quartet and doublet manifolds in a complex of Cr(III) for the first time. Two different luminescent quartet excited states are populated immediately following excitation, attributed to states of predominantly ^4^LMCT and ^4^MC character, the fluorescence decay from which allows us to assign a time constant to the intersystem crossing process of 823 fs, disentangled from other parallel excited-state events. This work highlights the power of combining both femtosecond TA and FLUPS, in conjunction with steady-state spectroscopies, in building up a comprehensive picture of the excited-state landscape of **1**^**3+**^ (Figure 5). We believe that such detailed photophysical studies and the resultant greater understanding of processes occurring throughout the excited-state landscape will be of significant value to those working to develop ever more efficient luminophores and photo-driven systems based upon new ligand scaffolds and Cr(III) in general.

## Experimental Section

### General Methods

All reagents were obtained from Acros Organics, Sigma-Aldrich, and Fluorochem and used as received. Acetonitrile (MeCN) and dichloromethane (CH_2_Cl_2_) were distilled from CaH_2_, purged with dry N_2_ for a period of 15 min, and then stored over 4 Å molecular sieves under an atmosphere of dry N_2_. [Cr(MeCN)_4_][BF_4_]_2_ was prepared according to the literature procedure,^70^ rigorously excluding air and being stored in an argon-filled glovebox. All synthetic manipulations involving Cr(II) salts were carried out under an inert atmosphere of argon or N_2_ using standard Schlenk line techniques. NMR spectra were recorded on a Bruker Ascend 400 MHz spectrometer, with chemical shifts being reported relative to the residual solvent signal (CDCl_3_: ^1^H δ 7.26, ^13^C δ 77.16). High-resolution mass spectrometry was performed on an Agilent 6210 TOF instrument with a dual electrospray ionization source. UV–visible electronic absorption spectra were recorded on an Agilent Cary-60 instrument while luminescence spectra were collected on a Horiba Fluromax-4 spectrometer. Lifetime measurements were carried out by single photon counting on an Edinburgh Instruments mini-τ, equipped with a picosecond diode laser (404 nm, 56 ps). Luminescence quantum yields are reported relative to [Ru(bpy)_3_]^2+^ in either aerated MeCN (ϕ = 1.8%) or H_2_O (ϕ = 4.0%),^58^ with all complexes being excited at a single wavelength of common optical density. Quantum yields are thus determined from the ratio of integrated peak areas, with an assumed experimental uncertainty of ±10%. Cyclic Voltammetry measurements were conducted for 1.5 mmol dm^–3^ analyte solutions in dry, deaerated MeCN under an atmosphere of dry N_2_. ^*n*^BuN_4_PF_6_ was utilized as the supporting electrolyte with a solution concentration of 0.2 mol dm^–3^. Glassy carbon was employed as the working electrode while Pt wire was utilized as the counter alongside a Ag/AgCl reference electrode. All potentials are measured against the Fc^+^/Fc couple. Magnetic susceptibility measurements were performed using Evans’ method^52^ making use of a co-axial NMR tube containing the paramagnetic analyte (9.05 mmol dm^–3^) in a solution of *d*^3^-MeCN (580 μL) and ^*t*^BuOH (20 μL).

### Quantum Yield of Singlet Oxygen Production

Singlet oxygen was detected through measurement of the singlet oxygen emission band at ∼1275 nm. Complex **1**^**3+**^ dissolved in acetonitrile solution was excited with the third harmonic of a Q-Switched Nd:YAG laser (λ = 355 nm, ∼8 ns pulse length, laser model LS-1231M from LOTISII). The time-resolved signal of ^1^O_2_ luminescence at 1275 nm was detected by a liquid-nitrogen-cooled InGaAs photodiode of Ø 3 mm active area (J22D-M204-R03M-60-1.7, Judson Technologies). The output from the photodiode was coupled into a low-noise current amplifier (DLPCA-200, FEMTO Messtechnik GmbH). The amplifier output signal was recorded with a digital oscilloscope (TDS 3032B Tektronix) and transferred to a computer. To selectively detect the ^1^O_2_ emission, a high-contrast bandpass optical filter (1277 nm center wavelength, 28 nm FWHM, custom-made by Izovac, Belarus) was fitted in front of the InGaAs photodiode. To increase the light collection efficiency, a spherical broadband mirror was set behind the sample to reflect the NIR emission through the sample toward the detector.

The quantum yield of singlet oxygen production (Φ ^1^O_2_) was determined by comparing the initial amplitude of the emission signal of ^1^O_2_ generated when irradiating an air-equilibrated solution of **1**^**3+**^ and that of the standard (perinaphthenone, Φ ^1^O_2_ = 100% (MeCN)).^71^ The emission lifetime for ^1^O_2_ sensitized by **1**^**3+**^ and the standard must be similar (within the range 70–90 μs in MeCN) to confirm that ^1^O_2_ does not react with the photosensitizer in its ground state. The optical densities of the complex and a standard were matched at 355 nm, and the same solvent was used for both compounds. The experiments were performed at a series of excitation energies ranging from 10 to 80 μJ per pulse. The Φ ^1^O_2_ values were obtained in the low-energy limit while the intensity of the emission increases linearly with the laser power.

### Transient Absorption Spectroscopy

UV–vis transient absorption spectroscopy experiments were performed in the Lord Porter Ultrafast Laser Laboratory (ULS) at The University of Sheffield, using a Helios system (HE-VIS-NIR-3200) provided by Ultrafast Systems. A Ti:Sapphire regenerative amplifier (Spitfire ACE PA-40, Spectra-Physics) provides 800 nm pulses (40 fs FWHM, 10 kHz, 1.2 mJ). The 400 nm pump pulses (2.5 kHz, 0.2 μJ) were generated through frequency doubling of the amplifier fundamental. The pump was focused onto the sample to a beam diameter of ∼190 μm. The white light probe continuum (420–700 nm) was generated using a sapphire crystal and a portion of the amplifier fundamental. The intensity of the probe light transmitted through the sample was measured using a CMOS camera, with a resolution of 1.5 nm. Prior to the generation of the white light, the 800 nm pulses were passed through a computer-controlled optical delay line (DDS300, Thorlabs), which provides up to 8 ns of pump-probe delay. The instrument response function was approximated to be 100 fs (FWHM), based on the temporal duration of the coherent artifact signal from neat acetonitrile.

### Fluorescence Upconversion Spectroscopy

Fluorescence upconversion spectroscopy experiments were performed in the Lord Porter Ultrafast Laser Laboratory at the University of Sheffield, using a setup that has been previously described in detail elsewhere.^66,67,72^ Pertinent experimental information is as follows.

Excitation was provided by a Ti:Sapphire regenerative amplifier (Spitfire ACE PA-40, Spectra-Physics) generating 800 nm pulses (40 fs FWHM, 10 kHz, 1.2 mJ). The amplifier was seeded by 800 nm pulses (25 fs FWHM, 84 MHz) generated by a Ti:Sapphire oscillator (Mai Tai, Spectra-Physics). Both amplification stages of the Spitfire ACE were pumped by two Nd:YLF lasers (Empower, Spectra-Physics). Gate pulses were at 1320 nm (80 fs FWHM, 10 kHz, 60 μJ), whereas the 400 nm excitation (40 fs FWHM, 10 kHz, 0.3 μJ) was generated by frequency doubling a portion of the Ti:Sapphire amplifier 800 nm output.

The power of pump pulses was attenuated before the sample using a variable attenuation neutral density filter wheel, with pulses passing through a mechanical optical delay stage to give an experimental time window of 2.6 ns and temporal resolution of 1.67 fs. The pump pulses were focused by a lens (*f* = 200 mm, fused silica) onto the sample cuvette (silica, 1 mm pathlength) to a spot size diameter of ≤0.1 mm. The sample solution was agitated with a magnetic stirrer and/or flowed with a peristaltic pump (ColePalmer, Teflon loop) throughout the measurements.

Emission from the sample was collected in a forward-scattering geometry. The fluorescence was collected in a β-barium borate crystal (100 μm BBO crystal, EKSMA OPTICS) where it was upconverted by sum-frequency generation with the gate pulses. The upconverted fluorescence signal was spatially filtered and then focused using a concave mirror onto a fiber optic bundle (Ceram Optek). A homemade spectrograph was used to disperse the upconverted fluorescence signal onto a CCD detector (iDus 420 DU440A-Bu2, Andor).

### Single-Crystal X-ray Diffraction

Single crystals of **1**^**3+**^(PF_6_)_3_ were obtained from the slow vapor diffusion of diisopropylether into a concentrated MeCN solution. Diffraction data were collected under a stream of cold N_2_ at 150 K on a Bruker D8 Venture diffractometer equipped with a graphite monochromated Mo(Kα) radiation source. Solutions were generated using Patterson heavy atom or direct methods and fully refined by full-matrix least-squares on *F*^2^ data using SHELXS-97 and SHELXL software, respectively.^73^ Absorption corrections were applied based on multiple and symmetry-equivalent measurements using SADABS.^74^ The structure contained a rotationally disordered anion and positionally disordered acetonitrile solvent molecules. In both cases, the disorder was modeled over two positions using the PART instruction, with its own free variable, in the least-squares refinement.

### Computational Methods

Quantum chemical calculations were performed to complement the experimental results at the open-shell unrestricted density functional theory (uDFT) and associated time-dependent DFT (TD-DFT) computational levels of theory. All calculations were performed using the B3LYP* hybrid functional^75^ which comprises a modification of the standard B3LYP functional with reduced (15%) Hartree–Fock exchange together with a standard 6-311G(d) triple-zeta basis set,^76,77^ that has been previously employed for several related transition-metal complexes.^78^ All calculations were furthermore performed with the program standard self-consistent reaction field (SCRF) model for an acetonitrile (CH_3_CN) solvent environment. Finally, a superfine integral grid was used throughout for computational reliability. Full optimizations were first performed for the lowest doublet and quartet multiplicity states. Subsequent calculations to assess the excited-state energy landscape included both single-point cross-energies of the lowest doublet at the optimized quartet geometry and vice versa using uDFT as well as calculations of the 10 lowest quartet–quartet vertical excitations at the optimized quartet ground state geometry using time-dependent DFT (TD-DFT). All calculations were performed using the Gaussian16 program.^79^

### Synthesis

**Caution!** Care should be exercised when preparing triazole-containing compounds utilizing organic azides as these precursors are potentially explosive. It is recommended that organic azides are not isolated but rather generated and used immediately *in situ*.

#### 2,6-*bis*(4-Phenyl-1,2,3-triazol-1-yl-methyl)pyridine (btmp)

A mixture of *N*,*N*-dimethylformamide (48 mL) and H_2_O (12 mL) was added to 2,6-*bis*(bromomethyl)pyridine (2.00 g, 7.55 mmol), sodium azide (1.03 g, 15.85 mmol), phenylacetylene (1.74 mL, 1.62 g, 15.85 mmol), potassium carbonate (1.15 g, 8.30 mmol), copper(II) sulfate pentahydrate (0.80 g, 3.17 mmol), and sodium ascorbate (1.26 g, 6.34 mmol). The mixture was stirred for 16 h. at r.t., forming a green suspension. CH_2_Cl_2_ (80 mL), H_2_O (70 mL), and conc. aq. NH_3_ (15 mL) were added, and the mixture was stirred vigorously at r.t. for 1 h. The organic layer was separated and the aqueous layer was extracted three times with CH_2_Cl_2_ (3 × 30 mL). The combined organic layers were washed twice with dilute ammonium hydroxide solution (2 × 70 mL) followed by H_2_O (70 mL) and then sat. brine (70 mL). The solution was dried over MgSO_4_, filtered, and all volatiles were removed *in vacuo* affording a pale yellow solid. The solid was suspended in MeCN (10 mL), sonicated, filtered, and then washed with MeCN followed by Et_2_O to give the title compound as a white solid. Yield: 2.77 g, 93%. ^1^H NMR (CDCl_3_, 400 MHz): δ 5.70 (s, 4H), 7.21 (d, *J* = 7.8 Hz, 2H), 7.32 (t, *J* = 7.4 Hz, 2H), 7.39 (t, *J* = 7.6 Hz, 4H), 7.71 (t, *J* = 7.8 Hz, 1H), 7.81 (d, *J* = 8.0 Hz, 4H), 7.87 (s, 2H). ^13^C NMR (CDCl_3_, 100 MHz): δ 55.66, 120.50, 122.29, 126.05, 128.61, 129.20, 130.69, 139.09, 148.67, 155.06. HRMS (ESI^+^); Calc’d for C_23_H_19_N_7_Na (M – Na^+^): *m*/*z* = 416.1600; Found: *m*/*z* = 416.1596. Anal. Calc’d for C_23_H_19_N_7_ (%): C 70.21, H 4.87, N 24.92, found (%): C 70.15, H 4.72, N 24.64.

#### [Cr(btmp)_2_][BF_4_]_3_ (**1**^3+^)

A Schlenk flask was charged with btmp (0.30 g, 0.76 mmol) and [Cr(MeCN)_4_][BF_4_]_2_ (0.15 g, 0.38 mmol). To the solids was added, by cannula, a 2:1 (v/v) mixture of dry, degassed MeCN and CH_2_Cl_2_ (10 mL) instantly giving a dark green solution. After stirring for 1 h. at r.t., AgBF_4_ (0.08 g, 0.40 mmol) was added and the reaction vessel opened to air. The resultant yellow-colored mixture was filtered, and the filtrate was concentrated to 2 mL *in vacuo*. Et_2_O (200 mL) was added with rapid stirring, forming a bright-yellow precipitate. The solids were collected by filtration, washed thoroughly with CH_2_Cl_2_ and Et_2_O, and then dried *in vacuo*, affording the product as a bright yellow solid. Yield: 0.31 g, 74%. HRMS (ESI^+^). Calc’d for CrC_46_H_38_N_14_B_2_F_8_ ([**1**]^3+^[BF_4_]^–^_2_)^+^: *m*/*z* = 1012.2861. Found: *m*/*z* = 1012.2864, Calc’d for CrC_46_H_38_N_14_ ([**1**]^3+^): *m*/*z* = 279.4264. Found: *m*/*z* = 279.4264. Anal. Calc’d for CrC_46_H_38_N_14_B_3_F_12_ (%): C 50.26, H 3.48, N 17.84, found (%): C 49.75, H 3.49, N 17.38.

## Supporting Information Available

NMR, mass spectrometry, and electrochemical data; additional spectroscopic data; and results from computational calculations (PDF)
