# Supplementary material for: Direct Determination of the Rate of Intersystem Crossing in a Near-IR Luminescent Cr(III) Triazolyl Complex
Source: J Am Chem Soc. 2023 May 24;145(22):12081–92. doi: 10.1021/jacs.3c01543 (PMC10251520; doi:10.1021/jacs.3c01543)
Supplement: Supplementary file 1 — ja3c01543_si_001.pdf [file ja3c01543_si_001.pdf]

Supporting Information for

Direct Determination of the Rate of Intersystem Crossing in a Near-IR Luminescent Cr(III) Triazolyl Complex

Robert W. Jones,[a] Alexander J. Auty,[b] Guanzhi Wu,[b] Petter Persson,[c] Martin V. Appleby,[b] Dimitri Chekulaev,[b] Craig R. Rice,[a] Julia A. Weinstein,[b] Paul I. P. Elliott [a] and Paul A. Scattergood [a]*

a Department of Chemistry, University of Huddersfield, Queensgate, Huddersfield, HD1 3DH, UK

b Department of Chemistry, University of Sheffield, Brook Hill, Sheffield, S3 7HF, UK

c Division of Theoretical Chemistry, Department of Chemistry, Lund University, Box 124, SE-22100 Lund, Sweden

Contents

**Figure S1**	^{1}H NMR spectrum for the ligand btmp	S2
**Figure S2**	^{13}C NMR spectrum for the ligand btmp	S2
**Figure S3**	ESI mass spectrum for the complex $\mathbf{1}^{3+}$	S3
**Figure S4**	UV-Visible absorption spectrum recorded for an MeCN solution of btmp	S3
**Figure S5**	Cyclic Voltammogram recorded for an MeCN solution of $\mathbf{1}^{3+}$	S4
**Figure S6**	Excitation spectrum recorded for an MeCN solution of $\mathbf{1}^{3+}$	S4
**Figure S7**	UV-Vis. absorption spectra recorded for an MeCN solution of $\mathbf{1}^{3+}$ over 0-72 hours	S5
**Figure S8**	UV-Vis. absorption spectra recorded for an aqueous solution of $\mathbf{1}^{3+}$ over 0-72 hours	S5
**Figure S9**	Spectral output profile of 23W compact fluorescent lamp (CFL) source	S6
**Figure S10**	Photostability studies: UV-Vis. absorption spectra recorded for $\mathbf{1}^{3+}$ in MeCN	S6
**Figure S11**	Photostability studies: Photoluminescence spectra recorded for $\mathbf{1}^{3+}$ in MeCN	S6
**Figure S12**	Photostability studies: UV-Vis. absorption spectra recorded for $\mathbf{1}^{3+}$ in H_2O	S7
**Figure S13**	Photostability studies: UV-Vis. absorption spectra recorded for $\mathbf{1}^{3+}$ in 0.1M HCl(aq.)	S7
**Figure S14**	Steady-state fluorescence spectrum recorded for an MeCN solution of $\mathbf{1}^{3+}$	S8
**Figure S15**	Selected FLUPS kinetic and spectral data from measurement cycles 1-4	S8
**Figure S16**	Calculated spin density plots for optimised quartet and doublet states of $\mathbf{1}^{3+}$	S9
**Figure S17**	Optimised geometry and spin density plot for $\mathbf{1}^{2+}$ (quintet state)	S9
**Table S1**	Summarised results from quantum chemical calculations	S9
	TD-DFT calculations of quartet-quartet vertical excitations	S10
**Table S2**	Selected calculated molecular orbitals for the quartet ground state	S11
	Optimised quartet state geometry for $\mathbf{1}^{3+}$	S13
	Optimised doublet state geometry for $\mathbf{1}^{3+}$	S15
	Optimised quintet state geometry for $\mathbf{1}^{2+}$	S17
	Optimised triplet state geometry for $\mathbf{1}^{2+}$	S19

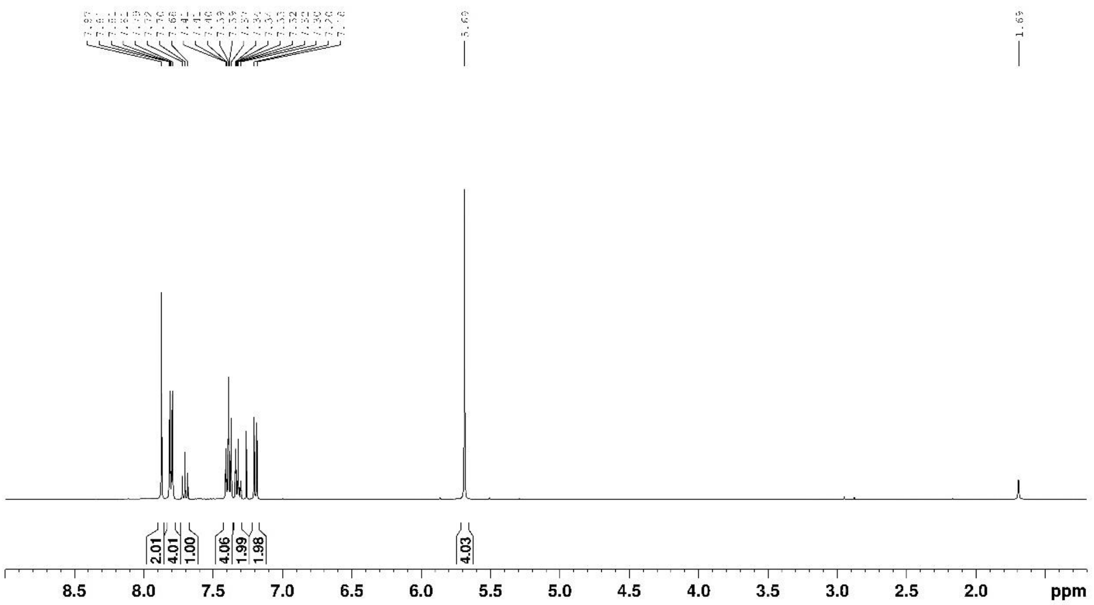

Figure S1 ^{1}H NMR (400 MHz, $CDCl_3$) spectrum of the ligand btmp.

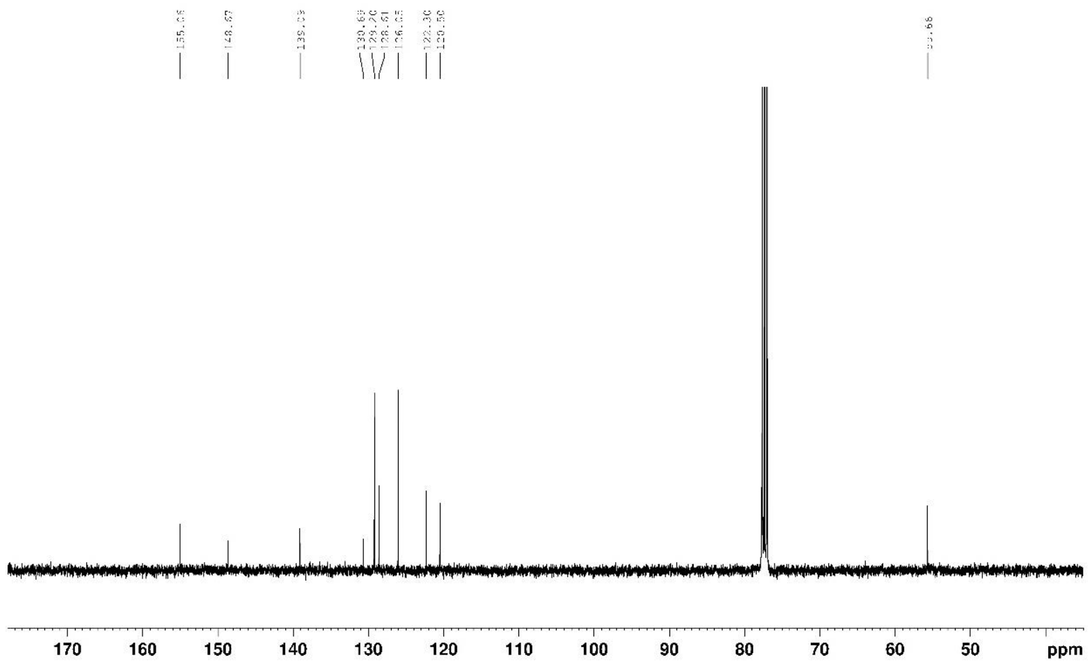

Figure S2 ^{13}C NMR ($CDCl_3$, 101 MHz) spectrum of the ligand btmp.

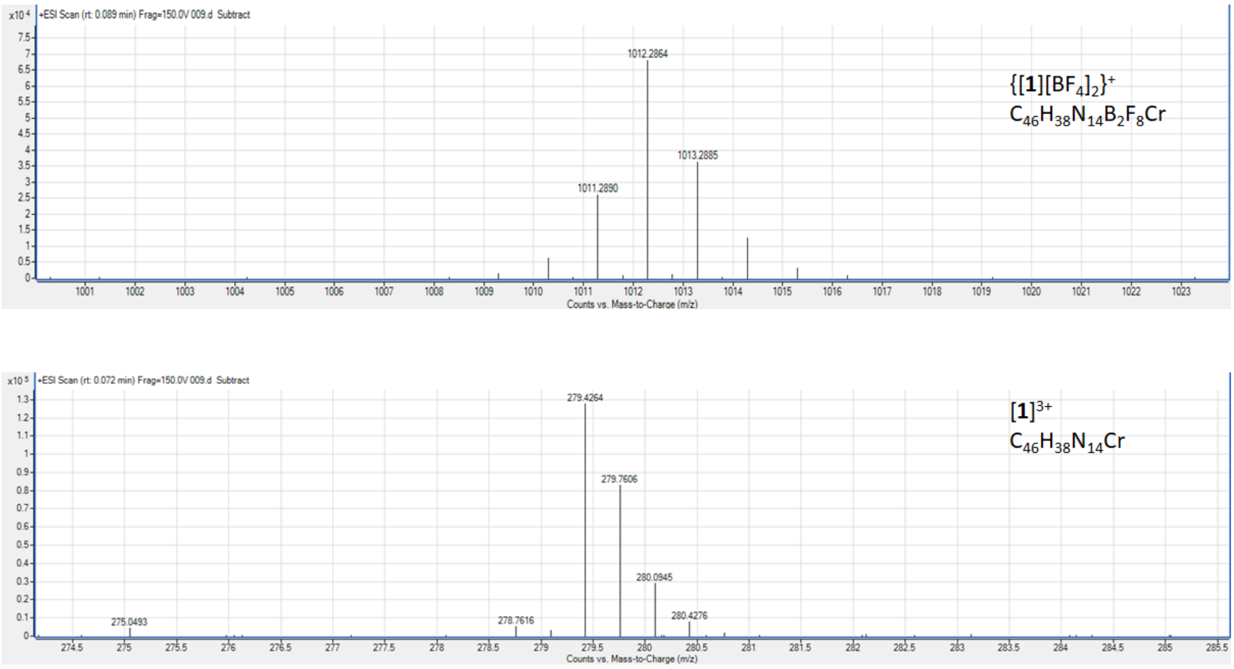

Figure S3 High Resolution ESI mass spectrum for $[Cr(btmp)_2][BF_4]_3$ (**1**), showing detail of the $\{[\mathbf{1}][BF_4]_2\}^+$ (top) and $[\mathbf{1}]^{3+}$ (bottom) mass fragments.

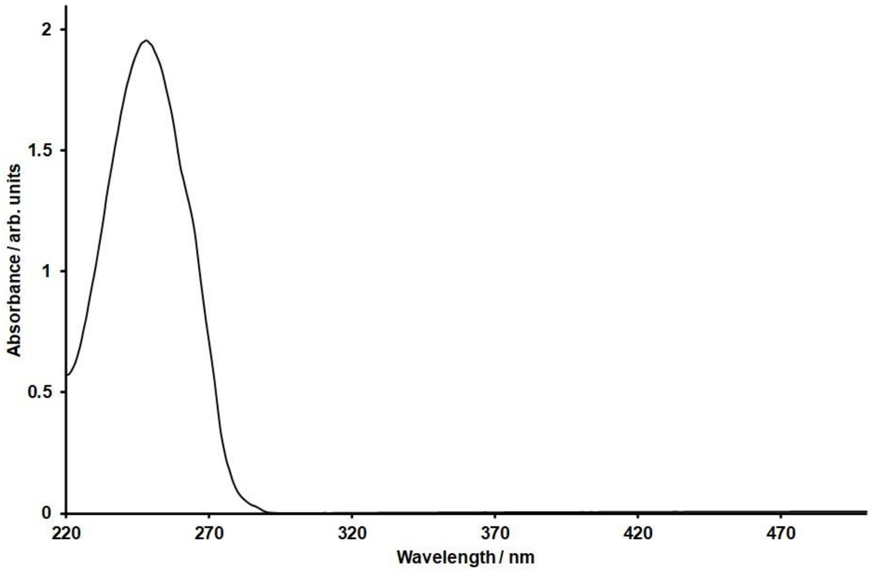

Figure S4 UV-Visible absorption spectrum recorded for an MeCN solution of the ligand btmp.

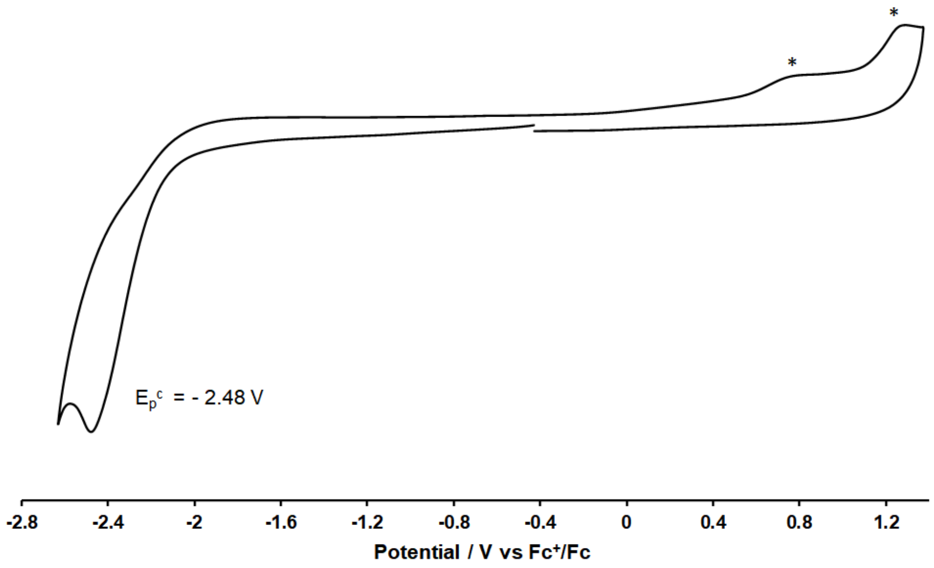

Figure S5 Cyclic voltammogram recorded at 100 mVs^{-1} for a 1.5 mmol dm^{-3} solution of $\mathbf{1}^{3+}$ in MeCN containing 0.2 mol dm^{-3} $^{n}NBu_4PF_6$. (Features marked * were also present in preliminary voltammograms recorded for the electrolyte-containing solvent in the absence of $\mathbf{1}^{3+}$).

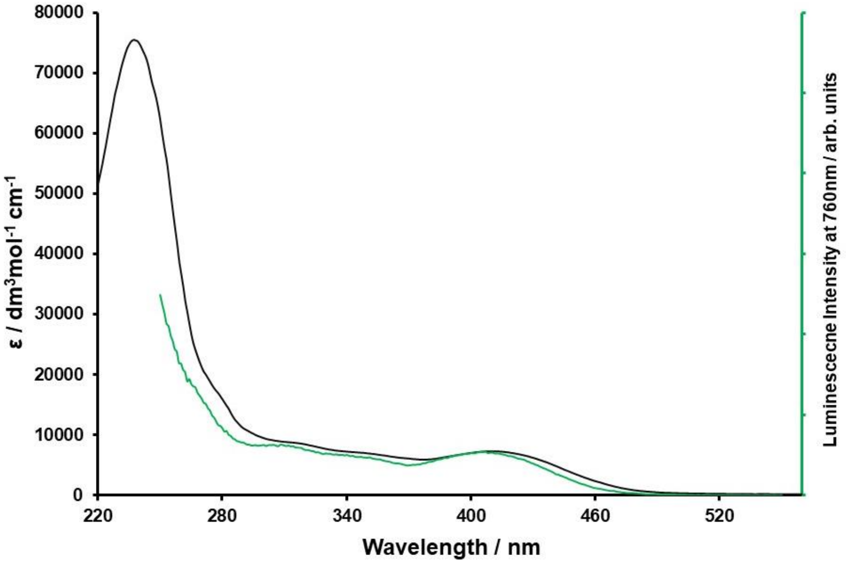

Figure S6 UV-Visible electronic absorption spectrum (black) and excitation spectrum for luminescence at λ_{em} = 760 nm (green) recorded for an aerated MeCN solution of $\mathbf{1}^{3+}$.

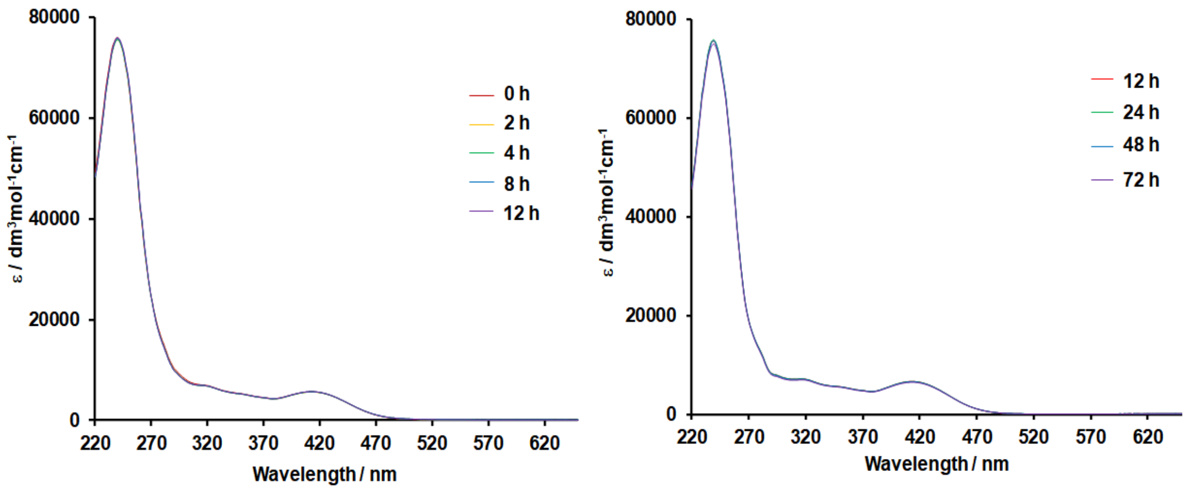

Figure S7 UV-Visible electronic absorption spectra recorded for an MeCN solution of **1**$^{3+}$, stored in the dark, over 0-72 hours.

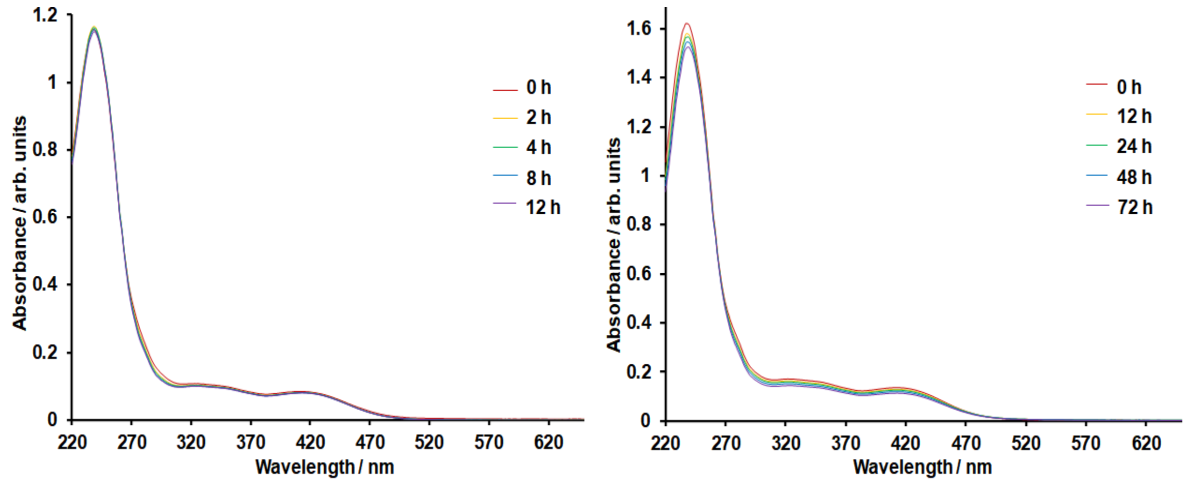

Figure S8 UV-Visible electronic absorption spectra recorded for an aqueous solution of **1**$^{3+}$, stored in the dark, over 0-72 hours.

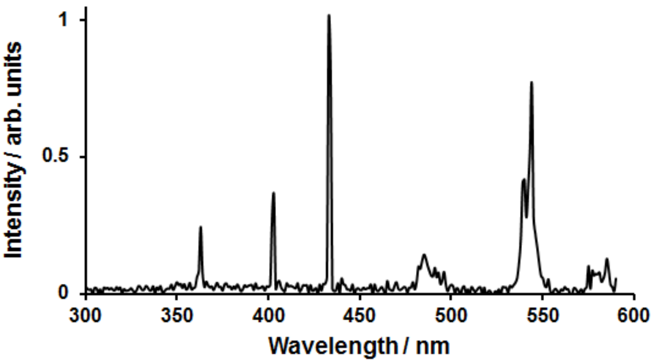

Figure S9 Output profile of the 23W compact fluorescent lamp (CFL) irradiation source employed for photostability experiments.

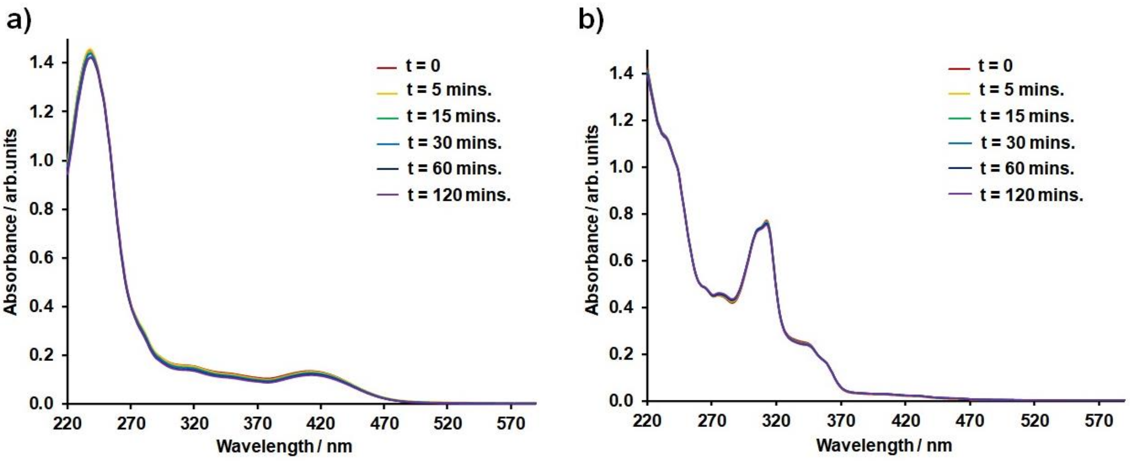

Figure S10 UV-Visible electronic absorption spectra recorded for MeCN solutions of $\mathbf{1}^{3+}$ (a) and $[Cr(bpy)_3][PF_6]_3$ (b) during irradiation with a 23W CFL.

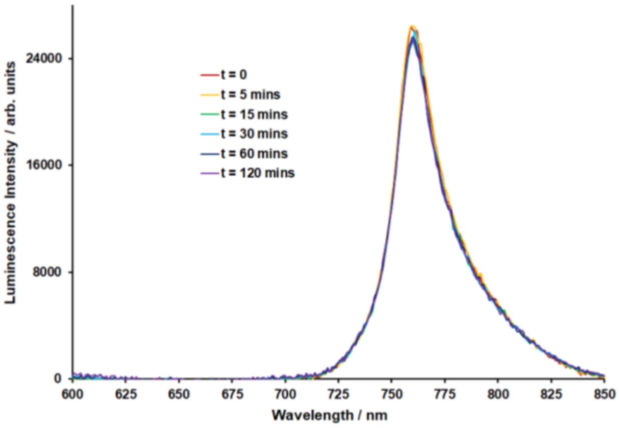

Figure S11 Photoluminescence spectra recorded for a room temperature aerated MeCN solution of $\mathbf{1}^{3+}$ during irradiation with a 23W CFL (λ_{ex} = 410 nm).

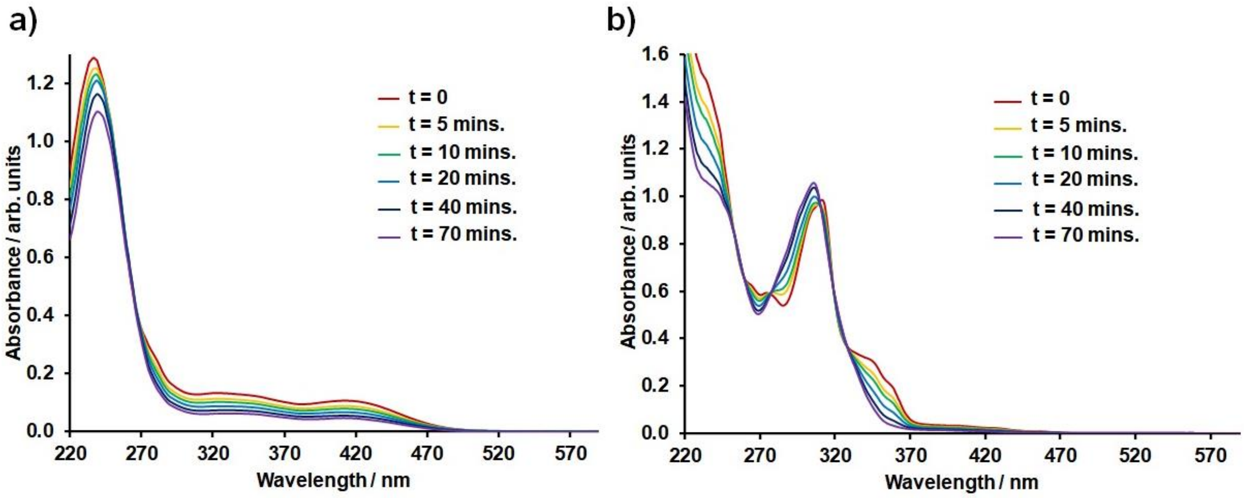

Figure S12 UV-Visible electronic absorption spectra recorded for aqueous solutions of **1**$^{3+}$ (a) and $[Cr(bpy)_3][PF_6]_3$ (b) during irradiation with a 23W CFL.

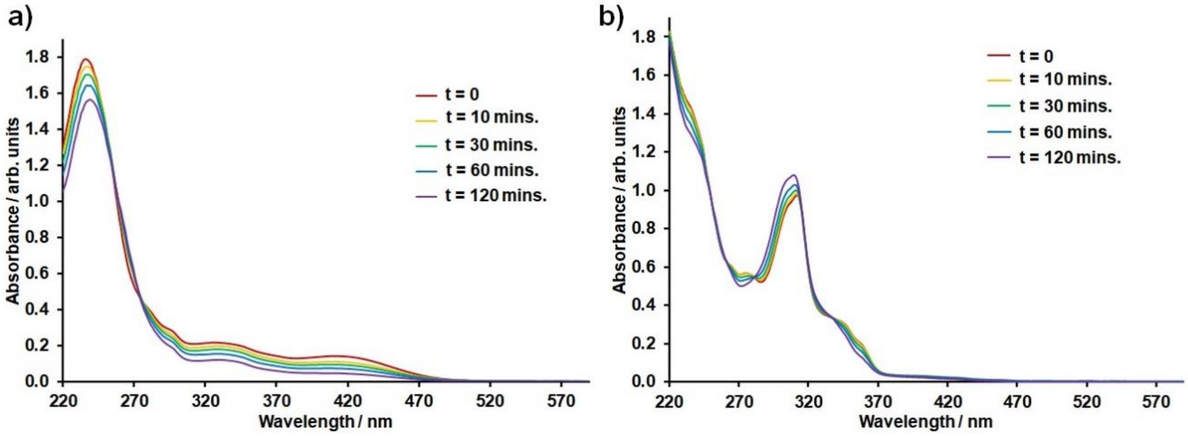

Figure S13 UV-Visible electronic absorption spectra recorded for 0.1M HCl(aq) solutions of **1**$^{3+}$ (a) and $[Cr(bpy)_3][PF_6]_3$ (b) during irradiation with a 23W CFL bulb.

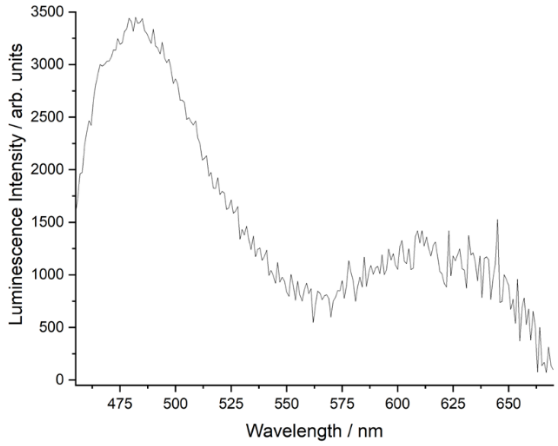

Figure S14 Steady-state fluorescence spectrum recorded for an 80 µM aerated room temperature MeCN solution of $\mathbf{1}^{3+}$ (λ_{ex} = 380 nm).

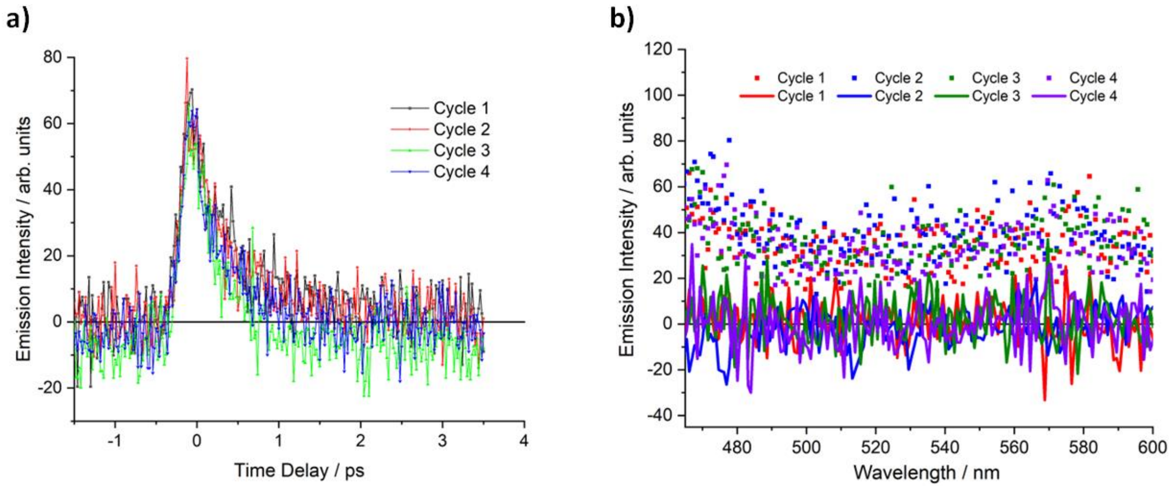

Figure S15 **a:** Kinetic decay traces at 550 nm obtained from each of the four FLUPS measurement cycles. **b:** Spectral data recorded at -500 fs (solid lines) and 140 fs (symbols) time delay for each of the four FLUPS measurement cycles. (These plots are presented to demonstrate the reproducibility of the FLUPS signal between cycles)

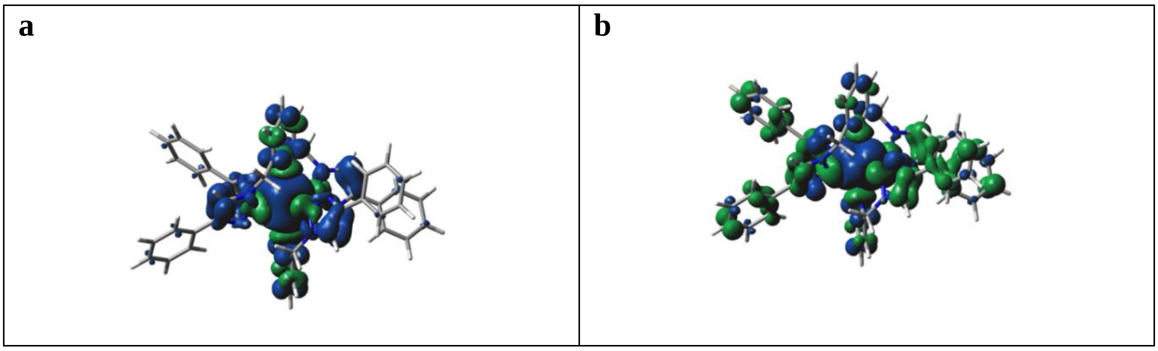

Figure S16 Calculated spin density plots for the optimized lowest energy quartet (a) and doublet (b) states of **1**$^{3+}$ (Results obtained at the uB3LYP*/6-311G(d) level of theory in acetonitrile).

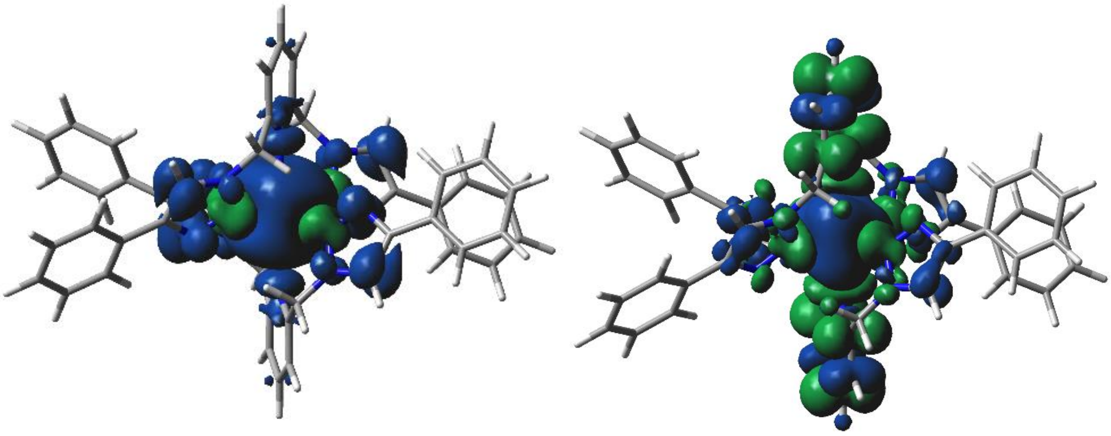

	^{5}**1**$^{2+}$	^{3}**1**$^{2+}$
Spin Density (Cr)	3.763282	2.319507
E_tot (au)	-3551.75285519	-3551.72812488
E_rel (eV)	0.00	0.67

Figure S17 DFT optimised geometry and calculated spin density plots for **1**$^{2+}$ (quintet state) (left) and **1**$^{2+}$ (triplet state) (right), together with calculated Mulliken spin densities and relative energies.

Table S1 Results from quantum chemical uDFT calculations for **1**$^{3+}$ carried out at the B3LYP*/6-311G(d) level of theory in an implicit acetonitrile solvent. For each state and geometry combination, the results include the total energies (E_{tot}) in Hartrees, the energy relative to the calculated overall ^{4}Cr(III) ground state (E_{rel}) in eV, as well as the calculated Mulliken Spin Density (SD) on the metal centre.

**Geometry**	**State**	**E_{tot} / Ha**	**E_{rel} / eV**	**SD_{Mull}(Cr)**
^{4}Cr(III)	^{4}Cr(III)	-3551.63169772	0.00	3.131289
	^{2}Cr(III)	-3551.55733134	2.02	1.395956
^{2}Cr(III)	^{2}Cr(III)	-3551.55939784	1.97	1.450503
	^{4}Cr(III)	-3551.62969966	0.05	3.112829

TD-DFT calculations of quartet-quartet vertical excitations.

The energy, oscillator strength (f) and most dominant orbital configurations are listed for each excitation (corresponding molecular orbitals are displayed in Table 2).

Excited State	1:	4.347-A	2.1757 eV	569.86 nm	f=0.0000	<S**2>=4.474	217→219 (0.90737)
							218→219 (0.14852)
Excited State	2:	4.403-A	2.2240 eV	557.48 nm	f=0.0003	<S**2>=4.596	216→219 (0.83162)
							215→219 (0.34175)
							217→219 (0.21248)
Excited State	3:	4.449-A	2.2608 eV	548.41 nm	f=0.0001	<S**2>=4.698	218→219 (0.67448)
							216→219 (0.33963)
Excited State	4:	4.398-A	2.2696 eV	546.29 nm	f=0.0002	<S**2>=4.586	215→219 (0.60974)
							218→219 (0.34360)
Excited State	5:	4.350-A	2.3084 eV	537.10 nm	f=0.0001	<S**2>=4.480	218→219 (0.58395)
							217→220 (0.58341)
Excited State	6:	4.248-A	2.4025 eV	516.07 nm	f=0.0330	<S**2>=4.260	215→217 (0.74823)
Excited State	7:	4.255-A	2.4145 eV	513.50 nm	f=0.0649	<S**2>=4.276	215→216 (0.55116)
Excited State	8:	4.261-A	2.4360 eV	508.96 nm	f=0.0415	<S**2>=4.288	213→217 (0.82459)
							215→218 (0.22359)
							215→220 (0.10786)
Excited State	9:	4.300-A	2.4680 eV	502.37 nm	f=0.0004	<S**2>=4.372	213→216 (0.75797)
Excited State	10:	4.175-A	2.4789 eV	500.15 nm	f=0.0041	<S**2>=4.107	215→216 (0.68691)
							214→217 (0.54582)

Table S2 Selected molecular orbitals for the quartet ground state

MO	Alpha	Beta
220	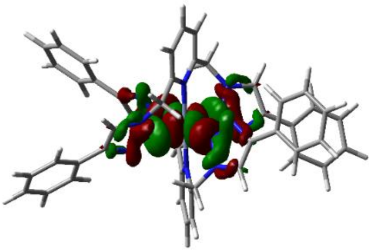	
219	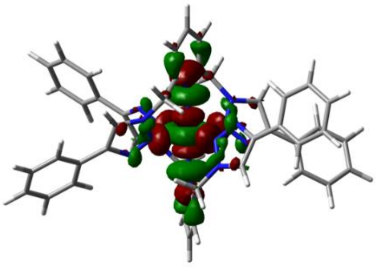 **Alpha-LUMO**	
218	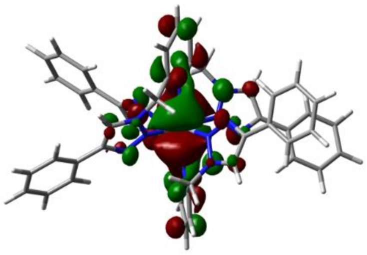 **Alpha-HOMO / SOMO-3**	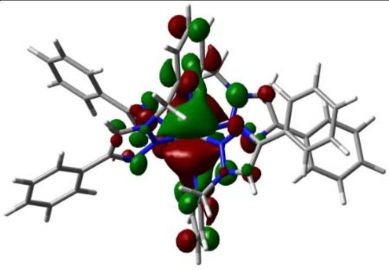 **SUMO-3**
217		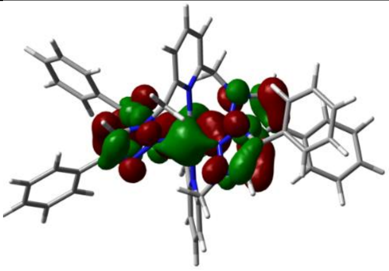 **SUMO-2**
216	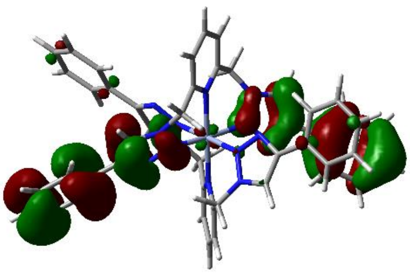	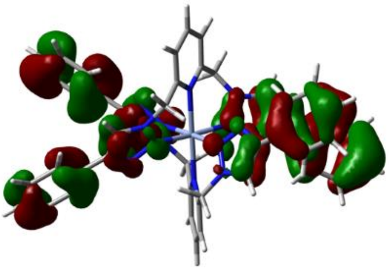 **Beta-LUMO / SUMO-1**

215	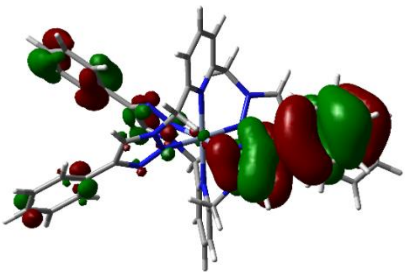	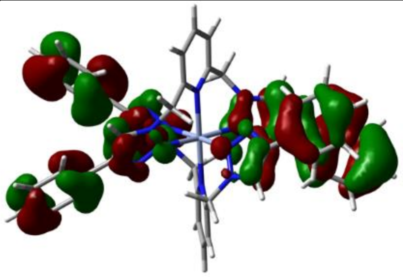 **Beta-HOMO**
214	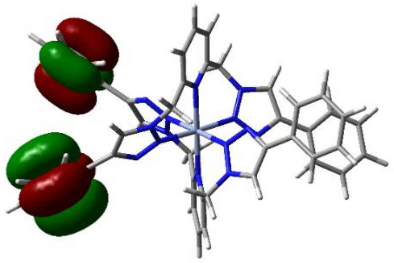	
213	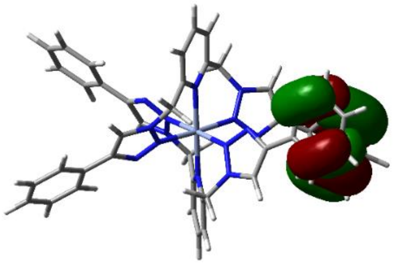	
186	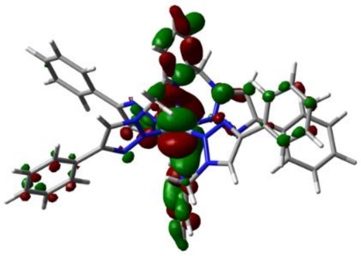	

Optimized quartet state geometry for 1^{3+}

Cr	0.000547	-0.038046	-0.002919
C	0.378599	0.957408	2.931149
C	-0.371282	-1.280923	2.833915
C	0.415007	0.910510	4.337711
C	-0.373153	-1.368152	4.239008
C	0.031314	-0.263524	5.006718
H	0.737783	1.797713	4.901624
H	-0.686686	-2.304472	4.722888
H	0.045842	-0.316025	6.106025
C	0.387164	-1.070227	-2.919609
C	-0.368506	1.167782	-2.855116
C	0.396501	-1.052714	-4.327281
C	-0.403350	1.222834	-4.261308
C	-0.011443	0.102942	-5.013292
H	0.717506	-1.948531	-4.878393
H	-0.729825	2.147164	-4.759388
H	-0.022133	0.131117	-6.113537
N	1.529271	-1.452217	-0.075983
C	2.977790	-2.845030	-1.016627
C	3.488734	-2.375811	0.218610
H	3.361687	-3.547447	-1.762297
N	-1.511140	1.394991	-0.003907
C	-2.901646	2.912110	-0.832256
C	-3.427967	2.378987	0.369899
H	-3.264202	3.669006	-1.533801
N	1.510929	1.391251	0.096805
C	2.910512	2.836456	1.031137
C	3.429708	2.394376	-0.210457
H	3.275909	3.539841	1.784901
N	-1.527387	-1.457120	-0.027084
C	-3.486181	-2.363779	-0.377625
C	-2.968600	-2.916032	0.819788
H	-3.356290	-3.654254	1.527574
N	-0.003012	-0.128710	2.174723
N	0.012582	0.029319	-2.179231
C	-0.731334	-2.547701	2.044254
H	-1.134259	-3.311752	2.730307
H	0.164369	-2.980648	1.556386
N	-1.760851	-2.325506	0.998680
N	-2.565257	-1.468564	-0.855763
C	0.710422	2.296500	2.257227
H	-0.200698	2.759185	1.828766
H	1.116647	2.997306	3.005644
N	1.727114	2.189727	1.180509
N	2.535082	1.501066	-0.740735
C	0.746545	-2.391730	-2.224400
H	-0.150749	-2.862649	-1.776820
H	1.157008	-3.099624	-2.963871
N	1.769695	-2.245074	-1.159360
N	2.564896	-1.516586	0.753342
C	-0.698101	2.455631	-2.086370
H	-1.097293	3.211139	-2.783593
H	0.212248	2.881299	-1.620082

N	-2.539386	1.444238	0.834737
N	-1.719787	2.274098	-1.024901
C	-4.696317	2.697976	1.080142
C	-5.565125	3.713506	0.596350
C	-5.054828	1.984100	2.256570
C	-6.766188	4.006257	1.274990
H	-5.309552	4.285359	-0.310829
C	-6.255714	2.282061	2.931047
H	-4.389592	1.194462	2.639213
C	-7.115738	3.292798	2.443470
H	-7.430789	4.796691	0.888675
H	-6.522176	1.720905	3.842188
H	-8.055336	3.523502	2.972648
C	4.770263	-2.698277	0.902680
C	5.812662	-3.372615	0.211075
C	4.966656	-2.329233	2.261666
C	7.023985	-3.673510	0.867582
H	5.690491	-3.655968	-0.847402
C	6.180186	-2.630855	2.911747
H	4.163405	-1.809537	2.807137
C	7.212179	-3.304254	2.218521
H	7.825537	-4.195169	0.318835
H	6.319797	-2.341183	3.966580
H	8.160409	-3.540120	2.729721
C	-4.775892	-2.633290	-1.069544
C	-5.633072	-3.677457	-0.627464
C	-5.166119	-1.844477	-2.186382
C	-6.854769	-3.923542	-1.287415
H	-5.350615	-4.310798	0.229508
C	-6.387379	-2.096451	-2.842960
H	-4.510217	-1.031803	-2.535644
C	-7.236255	-3.135093	-2.396213
H	-7.509587	-4.737496	-0.934667
H	-6.679167	-1.476680	-3.707122
H	-8.191865	-3.329754	-2.910994
C	4.692513	2.765574	-0.905347
C	5.579988	3.722691	-0.342858
C	5.024592	2.163089	-2.150002
C	6.772677	4.067696	-1.011752
H	5.347518	4.207237	0.619662
C	6.217531	2.512273	-2.813822
H	4.344539	1.419920	-2.594505
C	7.095830	3.464941	-2.248192
H	7.451789	4.812073	-0.564052
H	6.462955	2.037166	-3.778265
H	8.028816	3.736622	-2.769569

Optimized doublet state geometry for 1^{3+}

Cr	-0.011571	0.000060	-0.000227
C	0.365717	1.332009	2.821528
C	-0.413817	-0.876641	2.990960
C	0.376412	1.469291	4.223683
C	-0.454398	-0.788098	4.396574
C	-0.047338	0.398726	5.028558
H	0.707812	2.415636	4.675817
H	-0.794454	-1.651364	4.987614
H	-0.059081	0.486984	6.125782
C	0.366851	-1.331390	-2.821926
C	-0.413360	0.877008	-2.991408
C	0.378369	-1.468407	-4.224083
C	-0.453118	0.788693	-4.397057
C	-0.045244	-0.397855	-5.029025
H	0.710112	-2.414639	-4.676194
H	-0.793306	1.651832	-4.988181
H	-0.056401	-0.485953	-6.126269
N	1.485159	-1.404230	0.073474
C	2.936087	-2.899817	-0.702860
C	3.422847	-2.330807	0.505944
H	3.327458	-3.675003	-1.368190
N	-1.500655	1.406052	-0.135409
C	-2.934292	2.798502	-1.110448
C	-3.413534	2.428470	0.176652
H	-3.321680	3.467778	-1.884221
N	1.484890	1.404405	-0.073814
C	2.935943	2.899599	0.703165
C	3.422937	2.330588	-0.505538
H	3.327040	3.674986	1.368434
N	-1.500444	-1.406335	0.134813
C	-3.413248	-2.428949	-0.176990
C	-2.933783	-2.798950	1.110042
H	-3.321194	-3.468053	1.883970
N	-0.017329	0.170794	2.194760
N	-0.016969	-0.170410	-2.195147
C	-0.760469	-2.228122	2.355599
H	-1.183896	-2.904541	3.117308
H	0.146747	-2.713313	1.943904
N	-1.763773	-2.140360	1.263488
N	-2.498253	-1.576086	-0.730389
C	0.735240	2.563712	1.986328
H	-0.160904	2.985688	1.489550
H	1.156133	3.348133	2.638497
N	1.750319	2.294428	0.935902
N	2.496341	1.423036	-0.939986
C	0.735942	-2.563371	-1.986959
H	-0.160365	-2.985773	-1.490957
H	1.157166	-3.347369	-2.639384
N	1.750631	-2.294462	-0.935947
N	2.496502	-1.422806	0.939860
C	-0.760950	2.228277	-2.356059
H	-1.184587	2.904533	-3.117828
H	0.145918	2.713951	-1.944180

N	-2.498478	1.575542	0.729834
N	-1.764226	2.140036	-1.264041
C	-4.655044	2.837033	0.878800
C	-5.601939	3.693047	0.249290
C	-4.912821	2.372053	2.199826
C	-6.776875	4.074487	0.927377
H	-5.429770	4.065268	-0.773671
C	-6.088808	2.756824	2.871217
H	-4.186220	1.709430	2.694829
C	-7.024865	3.608862	2.238837
H	-7.502552	4.737958	0.428906
H	-6.277295	2.390880	3.894080
H	-7.945076	3.908589	2.767219
C	4.680777	-2.609056	1.241907
C	5.712810	-3.390562	0.650655
C	4.870225	-2.089540	2.553884
C	6.903459	-3.648930	1.358655
H	5.597393	-3.791845	-0.369390
C	6.062755	-2.350854	3.255278
H	4.076442	-1.485437	3.020054
C	7.082749	-3.131530	2.661931
H	7.696111	-4.254328	0.889061
H	6.197316	-1.945954	4.271934
H	8.015110	-3.335479	3.214144
C	-4.655038	-2.837295	-0.878761
C	-5.601166	-3.694267	-0.249403
C	-4.913928	-2.371174	-2.199167
C	-6.776311	-4.075747	-0.927109
H	-5.428057	-4.067376	0.773088
C	-6.090095	-2.756022	-2.870196
H	-4.188022	-1.707661	-2.693990
C	-7.025302	-3.609156	-2.238033
H	-7.501320	-4.740079	-0.428809
H	-6.279396	-2.389267	-3.892619
H	-7.945656	-3.908934	-2.766134
C	4.681005	2.608728	-1.241292
C	5.714483	3.387133	-0.648484
C	4.869038	2.092463	-2.554780
C	6.905142	3.645699	-1.356392
H	5.600231	3.785713	0.372735
C	6.061638	2.353831	-3.256010
H	4.074213	1.490670	-3.022154
C	7.083040	3.131503	-2.661116
H	7.698863	4.248741	-0.885583
H	6.195143	1.951368	-4.273772
H	8.015396	3.335598	-3.213287

Optimised quintet state geometry for 1^{2+}

Cr	0.001462	-0.001504	0.000007
C	0.329785	1.024912	3.255804
C	-0.315429	-1.223675	3.190690
C	0.358042	1.020986	4.666646
C	-0.335474	-1.303672	4.599504
C	0.013694	-0.162657	5.348229
H	0.637812	1.930414	5.220335
H	-0.613498	-2.243736	5.100466
H	0.016801	-0.195393	6.449245
C	0.332960	-1.032797	-3.255072
C	-0.314504	1.215164	-3.193607
C	0.358603	-1.031910	-4.665973
C	-0.337312	1.292133	-4.602519
C	0.011530	0.149841	-5.349455
H	0.638542	-1.942178	-5.218207
H	-0.616936	2.230963	-5.104909
H	0.012539	0.180212	-6.450544
N	1.544622	-1.497415	-0.310283
C	2.912252	-2.835807	-1.455333
C	3.517240	-2.461408	-0.238769
H	3.231854	-3.483391	-2.276772
N	-1.539873	1.510916	-0.232588
C	-2.902182	2.911343	-1.307641
C	-3.513413	2.469595	-0.116907
H	-3.221815	3.594168	-2.099948
N	1.541699	1.495703	0.313655
C	2.901501	2.843190	1.457089
C	3.507930	2.472765	0.239991
H	3.223076	3.481962	2.284568
N	-1.539758	-1.514253	0.228667
C	-3.513227	-2.472420	0.109946
C	-2.903277	-2.915936	1.300599
H	-3.225959	-3.596966	2.093253
N	0.005605	-0.078789	2.529734
N	0.008755	0.072016	-2.530768
C	-0.593325	-2.473945	2.339677
H	-0.884384	-3.324279	2.981805
H	0.319027	-2.768297	1.783852
N	-1.687471	-2.303473	1.339053
N	-2.636126	-1.612790	-0.509577
C	0.599657	2.324987	2.480386
H	-0.316573	2.648079	1.947203
H	0.890037	3.135233	3.172471
N	1.690211	2.220528	1.466778
N	2.633230	1.644005	-0.423431
C	0.606700	-2.330550	-2.477044
H	-0.308266	-2.654989	-1.942556
H	0.899103	-3.141502	-3.167476
N	1.697481	-2.219879	-1.464452
N	2.637456	-1.639322	0.426224
C	-0.591730	2.466850	-2.344526
H	-0.881911	3.316442	-2.988016
H	0.320670	2.761140	-1.788765

N	-2.636796	1.610830	0.504576
N	-1.686596	2.298354	-1.344288
C	-4.844306	2.806731	0.461026
C	-5.708342	3.733319	-0.183750
C	-5.271346	2.200871	1.674627
C	-6.967145	4.044113	0.371406
H	-5.403381	4.221107	-1.124379
C	-6.530052	2.515587	2.226234
H	-4.611320	1.480562	2.182737
C	-7.383481	3.437368	1.577905
H	-7.625209	4.765107	-0.141960
H	-6.846717	2.036577	3.168033
H	-8.368141	3.681698	2.010566
C	4.841661	-2.838067	0.329272
C	5.808390	-3.519241	-0.459463
C	5.158544	-2.517503	1.677945
C	7.058043	-3.874751	0.089694
H	5.594960	-3.768567	-1.512185
C	6.409829	-2.873006	2.221683
H	4.417245	-1.990254	2.298714
C	7.364100	-3.553759	1.431490
H	7.797440	-4.401953	-0.536199
H	6.639946	-2.618680	3.270059
H	8.342087	-3.831848	1.858895
C	-4.844793	-2.806792	-0.467841
C	-5.676281	-3.790729	0.133192
C	-5.304988	-2.140984	-1.637049
C	-6.936755	-4.097802	-0.420263
H	-5.342365	-4.328642	1.035912
C	-6.564816	-2.452829	-2.187773
H	-4.670403	-1.375663	-2.110600
C	-7.386398	-3.430979	-1.582290
H	-7.569274	-4.864031	0.058529
H	-6.907847	-1.926623	-3.094490
H	-8.372165	-3.672646	-2.013928
C	4.833546	2.850023	-0.324792
C	5.670660	3.787747	0.339029
C	5.282317	2.272944	-1.544526
C	6.924998	4.136973	-0.203206
H	5.346441	4.256014	1.283224
C	6.536218	2.626480	-2.083491
H	4.643430	1.543869	-2.066971
C	7.363156	3.558649	-1.415941
H	7.561910	4.866513	0.324550
H	6.870274	2.169276	-3.030124
H	8.344156	3.833307	-1.838648

Optimised triplet state geometry for 1^{2+}

Cr	-0.001097	-0.004379	-0.000545
C	0.331028	1.109129	2.889896
C	-0.341131	-1.145678	2.878141
C	0.350455	1.124809	4.297295
C	-0.363266	-1.175081	4.285350
C	-0.007030	-0.028726	5.017695
H	0.640819	2.046806	4.823360
H	-0.654684	-2.102179	4.801760
H	-0.007918	-0.034173	6.118477
C	0.340807	-1.118713	-2.889850
C	-0.336042	1.134625	-2.881299
C	0.361545	-1.136014	-4.297260
C	-0.357162	1.162325	-4.288493
C	0.001825	0.015749	-5.019228
H	0.654418	-2.058023	-4.821918
H	-0.649912	2.088235	-4.806275
H	0.001277	0.019796	-6.120018
N	1.555715	-1.429796	-0.076452
C	2.931578	-2.905328	-1.023825
C	3.506673	-2.403202	0.161996
H	3.260231	-3.646179	-1.758647
N	-1.555837	1.424653	-0.068737
C	-2.917914	2.923200	-0.999227
C	-3.497359	2.414740	0.182047
H	-3.245646	3.663517	-1.734808
N	1.551356	1.426411	0.079798
C	2.914264	2.913646	1.026953
C	3.494322	2.416469	-0.158817
H	3.242231	3.645880	1.770481
N	-1.554910	-1.433596	0.063531
C	-3.497356	-2.420792	-0.190506
C	-2.920593	-2.930923	0.991118
H	-3.252641	-3.668280	1.727846
N	-0.004445	-0.014668	2.158308
N	0.002189	0.005127	-2.159887
C	-0.654241	-2.446795	2.129694
H	-1.000627	-3.217060	2.839972
H	0.247856	-2.841287	1.621592
N	-1.723038	-2.293668	1.109488
N	-2.619348	-1.505338	-0.724903
C	0.645549	2.417465	2.154742
H	-0.255607	2.816153	1.648430
H	0.989924	3.181061	2.873096
N	1.716937	2.274858	1.135551
N	2.617703	1.507027	-0.705053
C	0.658817	-2.424823	-2.152288
H	-0.241163	-2.825094	-1.645118
H	1.005828	-3.188812	-2.868974
N	1.729327	-2.275369	-1.133380
N	2.622139	-1.502130	0.709389
C	-0.651299	2.436337	-2.134754
H	-0.997555	3.205349	-2.846429
H	0.249987	2.832174	-1.626353

N	-2.621373	1.498461	0.717936
N	-1.721475	2.284063	-1.115608
C	-4.800329	2.739390	0.826737
C	-5.648723	3.748829	0.295334
C	-5.215357	2.037894	1.992090
C	-6.880867	4.046626	0.913882
H	-5.351638	4.312572	-0.604554
C	-6.447272	2.340045	2.607358
H	-4.567197	1.253078	2.412149
C	-7.285447	3.344455	2.071693
H	-7.527161	4.832805	0.488894
H	-6.754994	1.786114	3.510217
H	-8.249215	3.578878	2.554016
C	4.809052	-2.729851	0.806439
C	5.797993	-3.481504	0.115315
C	5.082149	-2.288884	2.130471
C	7.026704	-3.788534	0.736273
H	5.618484	-3.822579	-0.917852
C	6.312898	-2.595998	2.745826
H	4.322778	-1.706723	2.675741
C	7.289363	-3.347712	2.053058
H	7.784176	-4.371414	0.185931
H	6.509332	-2.248199	3.774002
H	8.251155	-3.587730	2.536574
C	-4.801507	-2.740481	-0.835151
C	-5.611823	-3.806736	-0.358168
C	-5.256024	-1.976863	-1.945396
C	-6.846604	-4.098049	-0.974602
H	-5.280372	-4.422574	0.494239
C	-6.489871	-2.273439	-2.559579
H	-4.637753	-1.147378	-2.322514
C	-7.290893	-3.333565	-2.077072
H	-7.462949	-4.929088	-0.592634
H	-6.828921	-1.670721	-3.418912
H	-8.256462	-3.563224	-2.558090
C	4.798359	2.745818	-0.798869
C	5.648145	3.747841	-0.255823
C	5.213119	2.056301	-1.971455
C	6.881359	4.050115	-0.870057
H	5.351308	4.302218	0.649934
C	6.446100	2.362885	-2.582378
H	4.563875	1.277312	-2.400596
C	7.285654	3.359860	-2.035107
H	7.528733	4.830413	-0.435972
H	6.753586	1.818265	-3.490971
H	8.250266	3.597741	-2.514043
